# Azvudine is a thymus-homing anti-SARS-CoV-2 drug effective in treating COVID-19 patients

**DOI:** 10.1038/s41392-021-00835-6

**Published:** 2021-12-06

**Authors:** Jin-Lan Zhang, Yu-Huan Li, Lu-Lu Wang, Hong-Qi Liu, Shuai-Yao Lu, Yong Liu, Ke Li, Bin Liu, Su-Yun Li, Feng-Min Shao, Kun Wang, Ning Sheng, Rui Li, Jin-Jin Cui, Pei-Chun Sun, Chun-Xia Ma, Bo Zhu, Zhe Wang, Yuan-Hao Wan, Shi-Shan Yu, Yongsheng Che, Chao-Yang Wang, Chen Wang, Qiangqian Zhang, Li-Min Zhao, Xiao-Zhong Peng, Zhenshun Cheng, Jun-Biao Chang, Jian-Dong Jiang

**Affiliations:** 1 State Key Laboratory of Bioactive Substance and Function of Natural Medicines, Institute of Materia Medica, Chinese Academy of Medical Science and Peking Union Medical College, Beijing, 100050 China; 2 Institute of Medicinal Biotechnology, Chinese Academy of Medical Science and Peking Union Medical College, Beijing, 100050 China; 3 Institute of Medical Biology, Chinese Academy of Medical Science and Peking Union Medical College, Kunming, 650031 Yunnan China; 4 Genuine Biotech. Inc., Pingdingshan, 467000 Henan China; 5 Department of Respiratory Medicine, Zhongnan Hospital of Wuhan University, Wuhan Research Center for Infectious Diseases and Cancer, Wuhan, 430071 Hubei China; 6 The First Affiliated Hospital of Henan University of Chinese Medicine, Zhengzhou, 450000 Henan China; 7 Department of Internal Medicine, Henan Provincial Peoples Hospital, Zhengzhou, 450003 Henan China; 8 Henan Key Laboratory of Organic Functional Molecule and Drug Innovation, School of Chemistry and Chemical Engineering, Henan Normal University, Xinxiang, 453007 Henan China; 9 National Clinical Research Center for Respiratory Diseases, Chinese Academy of Medical Science and Peking Union Medical College, Beijing, 100005 China; 10 School of Chemistry, Zhengzhou University, Zhengzhou, 450001 Henan China

**Keywords:** Infectious diseases, Drug development

## Abstract

Azvudine (FNC) is a nucleoside analog that inhibits HIV-1 RNA-dependent RNA polymerase (RdRp). Recently, we discovered FNC an agent against SARS-CoV-2, and have taken it into Phase III trial for COVID-19 patients. FNC monophosphate analog inhibited SARS-CoV-2 and HCoV-OC43 coronavirus with an EC_50_ between 1.2 and 4.3 μM, depending on viruses or cells, and selective index (SI) in 15–83 range. Oral administration of FNC in rats revealed a substantial thymus-homing feature, with FNC triphosphate (the active form) concentrated in the thymus and peripheral blood mononuclear cells (PBMC). Treating SARS-CoV-2 infected rhesus macaques with FNC (0.07 mg/kg, qd, orally) reduced viral load, recuperated the thymus, improved lymphocyte profiles, alleviated inflammation and organ damage, and lessened ground-glass opacities in chest X-ray. Single-cell sequencing suggested the promotion of thymus function by FNC. A randomized, single-arm clinical trial of FNC on compassionate use (*n* = 31) showed that oral FNC (5 mg, qd) cured all COVID-19 patients, with 100% viral ribonucleic acid negative conversion in 3.29 ± 2.22 days (range: 1–9 days) and 100% hospital discharge rate in 9.00 ± 4.93 days (range: 2–25 days). The side-effect of FNC is minor and transient dizziness and nausea in 16.12% (5/31) patients. Thus, FNC might cure COVID-19 through its anti-SARS-CoV-2 activity concentrated in the thymus, followed by promoted immunity.

## Introduction

Severe acute respiratory syndrome coronavirus 2 (SARS-CoV-2) belongs to the subfamily Orthocoronavirinae in the family of *Coronaviridae*. Its genome is enveloped and contains single-stranded (+) RNA of a size between 26 and 32 kb. Of the 16 nonstructural proteins of SARS-CoV-2, two function as protease and one as RNA-dependent RNA polymerase (nsp12).^1–3^ SARS-CoV-2 infection causes coronavirus disease 2019 (COVID-19) characterized by flu-like symptoms, including fever, cough, severe acute respiratory distress syndrome, and death, about 4–5% of cases.^4,5^ In vivo viral-immunological changes of COVID-19 include rapid viral replication, inflammatory response, and damage to the lymphatic system. ^6–9^ A recent study showed that the reduction in CD4+ and CD8+ cell counts in patients with COVID-19 closely correlated with disease progression; the disease severity was associated with host factors such as age, lymphocytopenia, and possible cytokine storm.^10^ Based on clinical outcomes, the machine learning tools of artificial intelligence have identified lymphocyte reduction as one of the three key indications that predict mortality more than 10 days in advance and with an accuracy of more than 90%.^11^ As the mortality rate in aged patients with COVID-19 is much higher than that in young- or middle-age population,^5^ a good immunity may be essential for recovering from SARS-CoV-2 infection.

Currently, the development of highly effective anti-COVID-19^1^ drugs is one of the major researches focuses. Initial efforts in this direction concentrated on the screening of known drugs. The most known potential candidates against COVID-19 are lopinavir/ritonavir (Kaletra, initially known as a protease inhibitor that interferes with the reproduction of human immunodeficiency virus),^12,13^ ribavirin (a nucleoside analog with broad-spectrum antiviral activity used to treat patients with SARS),^14^ chloroquine (an antimalarial drug active against COVID-19),^14^ remdesivir [a nucleoside analog that inhibited SARS-CoV and Middle East respiratory syndrome (MERS)-CoV in vivo and suppressed COVID-19 replication through inhibiting RNA-dependent RNA polymerases (RdRp),^12^ and favipiravir (a nucleoside analog that inhibits RNA viruses such as influenza and Ebola via its inhibition on RdRp),^14^ among others. Of the candidates, remdesivir has been approved by the United States and Japan FDA for treating COVID-19 in 2020, although its therapeutic efficacy is still debatable.

**Fig. 1:**
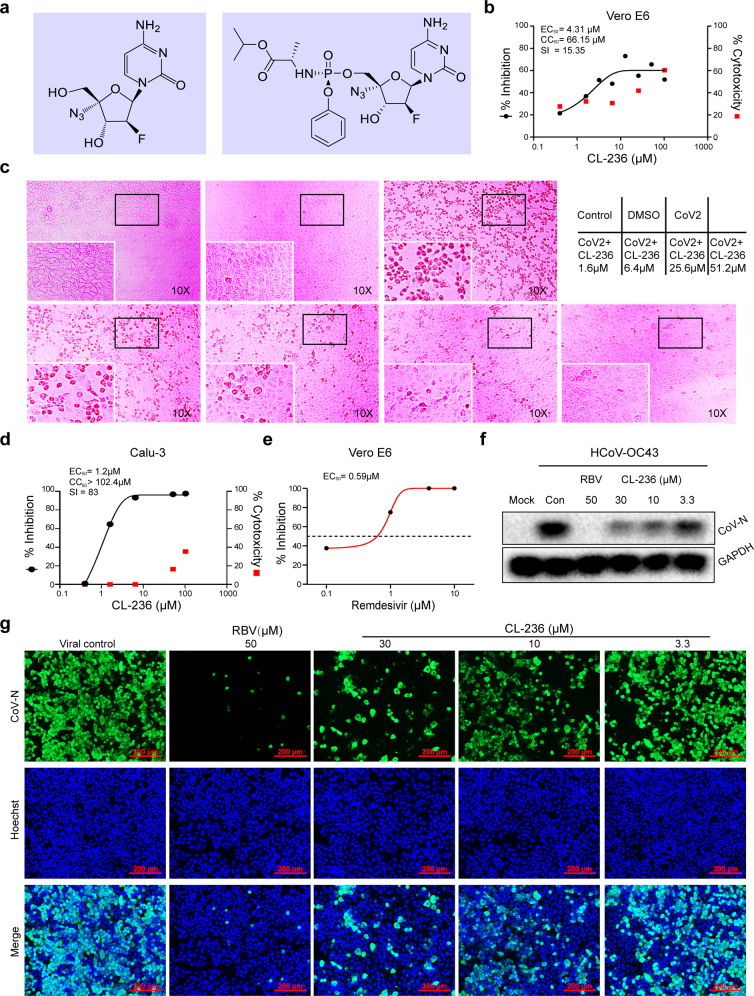
Anti-coronavirus activity of FNC analog CL-236 in vitro. Vero E6 cells and Calu-3 cells were pre-treated with the different doses of CL-236 for 1 h. SARS-CoV-2 (MOI = 0.05) was subsequently added to cells, followed by 1 h incubation. Then, the virus-drug mixture was removed and cells were further cultured with a fresh drug-containing medium for 48 h. Anti- SARS-CoV-2 efficacy of CL-236 was evaluated by measuring SARS-CoV-2 viral RNA copy numbers via qRT-PCR and CPE evaluation. For the antiviral test against HCoV-OC43, H460 cells were infected with HCoV-OC43 (MOI = 0.05). Then, various concentrations of CL-236 were added at the same time for 48 h incubation and then the CoV-N protein was analyzed using immunofluorescence analysis or determined by Western blot. **a** Chemical structure of FNC (left) and CL-236 (right). **b** CL-236 inhibited SARS-CoV-2 RNA replication in Vero E6 with EC_50_ of 4.3 μM; CC_50_ of 66.15 μM (qRT-PCR, 48 h). **c** CL-236 protected Vero E6 cells from SARS-CoV-2 infection caused CPE in 48 h. **d** CL-236 suppressed SARS-CoV-2 RNA replication in Calu-3 with EC_50_ of 1.2 μM; CC_50_ > 102.4 μM (qRT-PCR, 48 h). **e** Remdesivir inhibited SARS-CoV-2 in Vero E6 cells (48 h). **f** CL-236 decreased expression of HCoV-OC43 CoV-N protein in H460 cells (western blot, 48 h). **g** CL-236 reduced HCoV-OC43 CoV-N protein expression in H460 cells (immunofluorescence, 48 h). RBV ribavirin

Nucleoside analog 2′-deoxy-2′-β-fluoro-4′-azidocytidine, known as azvudine or FNC (MW, 286.22, Fig. 1a, left), is a prodrug that can be intracellularly converted into FNC triphosphate and inhibits viral RdRp.^15,16^ It has a broad-spectrum activity against viruses, including HCV and EV71,^15,16^ and is now an investigational drug against AIDS in China.^17^ FNC has been approved by China FDA for AIDS treatment on July 21, 2021 (XZXK-2021-214), showing efficacy in treating AIDS and good safety during the 48-week oral treatment. Recently, we discovered that oral administration of FNC could largely concentrate the drug in the thymus in its active form, efficiently inhibit SARS-CoV-2 replication in vivo, preserve thymus immune function, and rapidly cure patients with COVID-19 (patent pending). The following text described the therapeutic nature of FNC in laboratories, rhesus macaques (RM), as well as COVID-19 patients.

## Results

### FNC inhibited the replication of coronaviruses in vitro

FNC compound itself is almost inactive in inhibiting viral replication in vitro; it needs to be phosphorylated three times to be transferred to FNC triphosphate (FNC-TP), the active form of drug that inhibits RdRp of viruses.^15–17^ The phosphorylation of FNC occurs in the cytoplasm by deoxycytidine kinase with good efficiency.^15,16^ To better detect its anti-coronavirus activity in vitro, the analog of FNC monophosphate (CL-236, Fig. 1a right) was synthesized and used in the tests. It should be mentioned here that the reference drug remdesivir also contains phosphate for the same reason.

A conventional antiviral experiment was performed to learn the activity of CL-236 on host cell survival, viral replication, and cytopathic effect (CPE) of SARS-CoV-2. Vero E6 cells were first treated with the study drugs for 1 h and then infected with SARS-CoV-2 (BetaCoV/Wuhan/WIV04/2019) at a multiplicity of infection of 0.05. The DMSO solvent was used as a control. Anti-SARS-CoV-2 efficacy was then evaluated by measuring SARS-CoV-2 viral RNA copy numbers via quantitative real-time RT-PCR (qRT-PCR) and CPE 48 h post-infection (pi). As shown in Fig. 1b, CL-236 significantly inhibited viral replication (SARS-CoV-2 RNA copies) with a 50% effective dose (EC_50_) of 4.31 μM and selectivity index (SI) of 15.35. Original CPE results are demonstrated in Fig. 1c, showing that CL-236 protected the cells from SARS-CoV-2-caused cell death. The experiment was also performed in human lung adenocarcinoma Calu-3 cells using the same virus and experimental protocol. The EC_50_ of CL-236 in the Calu-3 cells was 1.2 μM and SI was 83 (Fig. 1d), showing an inhibitory effect better than that seen in the Vero E6 cells. Remdesivir was used as a positive reference in the Vero E6 experiment with an EC_50_ of 0.59 μM, stronger than that of CL-236 (Fig. 1e).

To learn the antiviral activity of CL-236 in other coronaviruses, H460 cells (human lung adenocarcinoma cells) were infected with HCoV-OC43 and treated with (or without) the drug at the same time. CL-236 significantly inhibited HCoV-OC43 infection, with an EC_50_ of 1.2 μM and SI of 20, evaluated by CPE. The results agreed with those obtained in SARS-CoV-2 infection. As shown in Fig. 1f, the HCoV-OC43 viral nucleoprotein (N protein) was examined by Western blot analysis, showing a decrease in N protein expression along with the increase in the CL-236 concentration. The anti-coronavirus effects were further verified by visualizing the viral N protein via immunofluorescence staining after 48 h (Fig. 1g). Ribavirin was used as a reference. The results validated the anti-coronavirus effect of CL-236 in vitro.

### FNC selectively activated through phosphorylation in the thymus in vivo

FNC achieved great success in treating patients suffering from COVID-19 during the SAR-CoV-2 outbreak 2020 in China (see results below); the clinical dose of FNC was 5 mg per day in oral administration, much lower than that of remdesivir (100 mg per day, iv) and favipiravir (1000 mg per day, oral), the efficiency of FNC in treating patients with COVID-19 as a regular viral RdRp inhibitor needed further exploration.

**Fig. 2:**
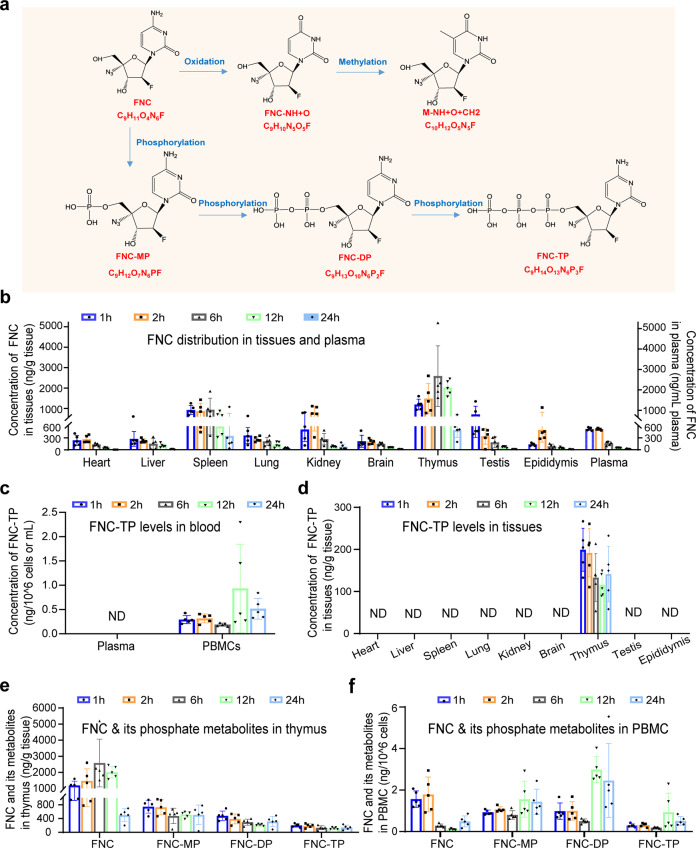
Thymus-homing distribution and metabolism of FNC after oral administration in rats. Twenty-five male Sprague–Dawley rats were orally administered with FNC at a single dose of 5 mg/kg. At the time point of 1, 2, 6, 12, and 24 h after drug administration, 5 rats were dissected. The blood, as well as the heart, liver, spleen, lung, kidney, brain, thymus, testis, and epididymis, were collected. Peripheral blood mononuclear cells (PBMCs) were isolated from blood using Histopaque-1083. The distribution of FNC in plasma, PBMCs, and the different tissues was evaluated through UHPLC–MS/MS analysis performed on an UHPLC system (1290 series, Agilent Technologies, USA) coupled to a triple quadrupole mass spectrometer (Agilent 6470 QQQ). **a** Metabolic pathway of FNC. **b** FNC distribution in tissues and plasma (5 mg/kg, oral). **c** FNC-TP levels in the blood (5 mg/kg, oral). **d** FNC-TP levels in tissues (5 mg/kg, oral). **e** FNC and its phosphate metabolites (FNC-MP, FNC-DP, and FNC-TP) in the thymus (5 mg/kg). **f** FNC and its phosphate metabolites in PBMCs (5 mg/kg). ND not detectable

Therefore, the in vivo distribution and phosphorylation of FNC was investigated after FNC was orally administered to the animals. FNC and its metabolites in rat organs were detected with an ultra-high-performance liquid chromatography coupled with a tandem mass spectrometer (UHPLC–MS/MS) (Supplementary Fig. 1). The in vivo metabolic pathway of FNC is shown in Fig. 2a and Supplementary Table 1, demonstrating the transformation route of FNC before it became active. As shown in Fig. 2b and Supplementary Table 1a, FNC was detected in the plasma with the peak level of about 670 ng/mL in the first 1 and 2 h after oral administration, whereas the FNC triphosphate was not detectable in the plasma (Fig. 2c and Supplementary Table 1b). The organ distribution showed that FNC was detectable in all organs tested, and the highest level in the first 2 h was found in the thymus and spleen; then, it was more concentrated in the thymus 6 h after oral administration, suggesting the FNC-enrichment effect in thymus tissues (Fig. 2b and Supplementary Table 1a). Interestingly, FNC triphosphate (FNC-TP) was seen only in the thymus, with all the organs showing the levels of FNC-TP below the detectable line (Fig. 2d and Supplementary Table 1c), indicating an inherent and steady transfer of FNC into its monophosphate, diphosphate, and triphosphate analogs in the thymus (Fig. 2e and Supplementary Table 1d). These phosphate metabolites of FNC were also detectable in peripheral blood mononuclear cells (PBMCs; Fig. 2f and Supplementary Table 1e), indicating good phosphorylation of FNC in PBMCs, which mainly consisted of lymphocytes and monocytes.

The chemical analysis of FNC intracellular metabolism also showed that FNC could be broken down into FNC-NH + O and FNC-NH + O + CH_2_ metabolites via pathways identical to that of natural 2′-deoxycytidine (Fig. 2a). The FNC-NH + O and FNC-NH + O + CH_2_ metabolites of FNC were indeed found in the thymus (Supplementary Fig. 2), verifying the thymus-homing feature of FNC. It appeared that the immune system, especially the thymus, was the target organ of FNC and FNC-TP.

In this study, remdesivir was detected and analyzed for comparison (Supplementary Fig. 3). The metabolic pathway of remdesivir is shown in Supplementary Fig 4a and Supplementary Table 2, demonstrating the essential transformation steps of the compound in vivo. As remdesivir was given intraperitoneally (ip), we detected remdesivir and its metabolites 2 and 6 h after ip injection. Although remdesivir and its metabolites Ala-Nuc and Nuc were detectable in most of the organs (Supplementary Fig. 4b–d and Supplementary Table 2a–c), the active compound remdesivir triphosphate (Nuc-TP) was detected in the lung, epididymis, and PBMCs (Supplementary Fig. 4h and Supplementary Table 2d–g). The level of Nuc-TP in the thymus was below the detectable line (Supplementary Fig 4e and Supplementary Table 2d), showing a distribution pattern quite different from that of FNC. A stable transformation from remdesivir to Nuc, Nuc-MP, Nuc-DP, and Nuc-TP was observed in the lung, PBMCs, and epididymis (Supplementary Fig. 4e–h and Supplementary Table 2e–g). It appeared that remdesivir might treat COVID-19 mainly through inhibiting SARS-CoV-2 in the lung (and epididymis), whereas FNC might cure COVIC-19 via inhibiting SARS-CoV-2 in the thymus, which might subsequently promote host immunity to fight SARS-CoV-2.

### Clinical benefits of FNC in RM infected with SARS-CoV-2

RM monkeys were used to learn whether FNC could inhibit SARS-CoV-2 in vivo. In this experiment, eight monkeys were first infected with SARS-CoV-2 using the protocol established previously with an infection dose of 10^6^ pfu per monkey.^18^ Among the eight animals, four were untreated (as vehicle control; two male and two female) and four were treated with FNC (0.07 mg/kg, qd, oral; two male and two female) 12 h post-infection. The treatment continued for 7 days from Day 1 to Day 7. The experiment was terminated on Day 8 after viral infection. Clinical manifestations, blood indications, and viral load in swabs and blood samples was monitored during the course, and organs were examined after euthanasia at 8 dpi. The experimental protocol is shown in Supplementary Fig. 5.

**Fig. 3:**
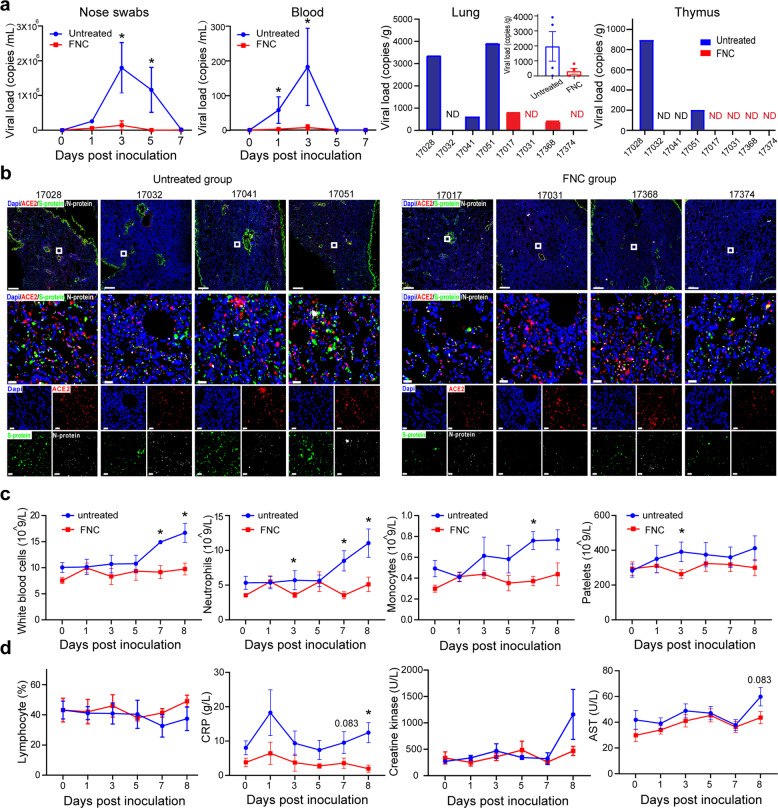
FNC inhibited SARS-CoV-2 and treated COVID-19 in vivo. Eight RM monkeys were inoculated with SARS-CoV-2, followed by vehicle or FNC treatment 12 h post infection and continued for 7 days (see Materials and methods). Viral load, hematology, immunology, blood biochemical, and histological evaluation were conducted at the indicated time points. **a** FNC significantly reduced viral load in nasal swabs, blood, as well as in lungs and thymus. **b** Representative images of multi-color immunofluorescent staining for ACE2 (red), S protein (green) and N protein (white) in lung tissues of RM monkeys inoculated with SARS-CoV-2, treated or untreated with FNC. The regions of interest (ROI) are boxed in white, and their magnified photos are shown below. Scale bars, 500 µm (up) and 20 µm (below). **c** White blood cells (WBC), neutrophil granulocytes (NG), monocytes (MC), and platelets basically remained stable by FNC after SARS-CoV-2 infection. **d** FNC increased the percent of lymphocytes, alleviated CRP production, and protected the heart and liver functions. Data are presented as mean ± SEM (*n* = 4); *p < 0.05, infected monkeys in FNC group vs. infected monkeys in the untreated group, by Mann–Whitney *U* test. CRP C reaction protein, AST aspartate aminotransferase

SARS-CoV-2 viral load of either nasal swabs or blood samples in the untreated monkeys was significantly higher than that in the FNC-treated ones (Fig. 3a), demonstrating a significant antiviral potency of FNC in vivo. The viral load in throat swab samples was only about 1% of that in the nasal swab samples in the RM model and was easily influenced by taking food or water, and thus it was not presented. The viral load in the lung at the end of the experiment was also detected. The viral load in the lung in four monkeys treated with FNC showed their viral load in the lung was lower than that in the four monkeys treated with vehicle (Fig. 3a). The observed insignificant statistics were probably due to the limited number of RM monkeys and a significant variation in the group.

The viral infection in the lung of each monkey was also examined with immune staining for ACE2 and viral S- and N-proteins, shown under low magnification (upper panel), high magnification (middle panel), and decomposition diagram (lower panel) (Fig. 3b). FNC noticeably reduced the viral S- (green) and N-protein (white) signal in the lung, consistent with the viral load results (Fig. 3a); in contrast, ACE2 signal (red) was almost at an equivalent level in the two groups.

During SARS-CoV2 infection, white blood cell (WBC) count, neutrophil count, monocyte count, platelet count, and the levels of C-reactive protein (CRP), creatine kinase (CK), and aspartate aminotransferase (AST) increased in patients and correlated with the severity and prognosis of the disease.^19–24^ Therefore, these parameters were analyzed in the monkeys. As shown in Fig. 3c and d, in the middle and late stages of FNC treatment, the WBC count, neutrophil count, monocyte count, platelet count, and CRP level in the untreated monkeys were significantly higher than those in the FNC-treated ones, suggesting a good control of viral infection by FNC in this model. In addition, FNC treatment decreased CK and AST levels, though insignificantly. More importantly, a moderate protective effect on lymphocyte counts was detected in the FNC-treated monkeys at the end of the experiment (Fig. 3d and 6). Other results of the blood test are shown in Supplementary Fig. 6.

**Fig. 4:**
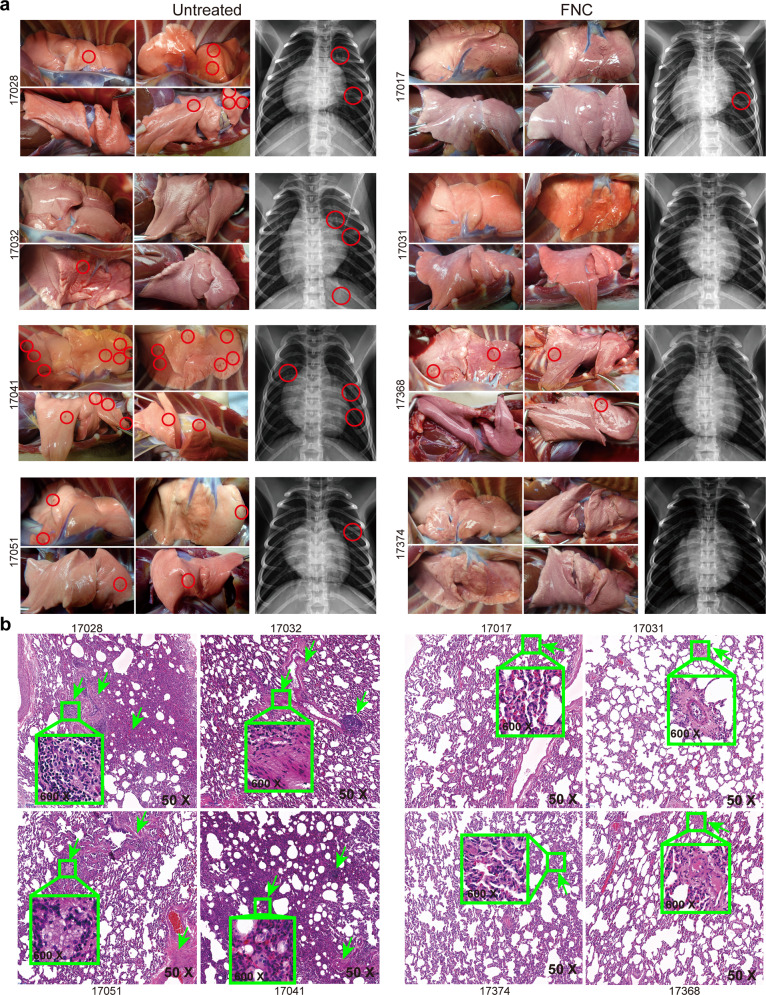
FNC protected lung from SARS-CoV-2 infection. **a** left. FNC reduced petechial spots (red circle) in lung of virus (+) monkeys. **a** right. Chest X-ray images clearly showed the ground-glass shadows or light shadows in the untreated monkeys (red circle), but much less in the FNC-treated virus (+) monkeys. **b** HE staining of the lung tissues was done and evaluated. Lesions in the lung of the untreated virus (+) monkeys included interstitial infiltration of neutrocytes or monocytes or macrophage, thickening of alveolar septae and vessel wall, blood effusion, edema, and fibrin with hyaline membranes as well as damage in cell structures (see green arrows); treating the monkeys with FNC substantially reduced the lesion described above

Furthermore, the lung was visually examined and histologically inspected. More petechial spots were seen in the lung of the untreated virus (+) monkeys than those of the FNC-treated ones (Fig. 4a left). Accordingly, HE staining demonstrated lesions in the lung of the untreated virus (+) monkeys, showing the interstitial infiltration of neutrophils or monocytes or macrophages, thickening of alveolar septae and vessel wall, blood effusion, edema and fibrin in hyaline membranes, and damage in cell structures (Fig. 4b); treating the monkeys with FNC reduced the lesions substantially (Fig. 4b). Chest X-ray imaging clearly showed the ground-glass opacities or light shadows in the untreated monkeys, much more than in the FNC-treated virus (+) monkeys (Fig. 4a right). However, body temperature and body weight changes after SARS-CoV-2 infection in the FNC-treated monkeys were not different from those in the untreated ones (Supplementary Fig. 7), probably because the disease was not severe and showed a self-limited course.^25^

### Thymus appeared to be very important in FNC’s mode of action against SARS-CoV-2

The viral load in the thymus was examined at the termination of the experiment. The four virus (+) monkeys treated with FNC showed a negative viral load. However, two out of four monkeys in the untreated group were highly positive for SARS-CoV-2 (Fig. 3a), suggesting a good antiviral effect of FNC in the thymus. The results were further supported by the good phosphorylation capacity for FNC in the monkey’s thymus (Supplementary Fig. 8 and 9).

**Fig. 5:**
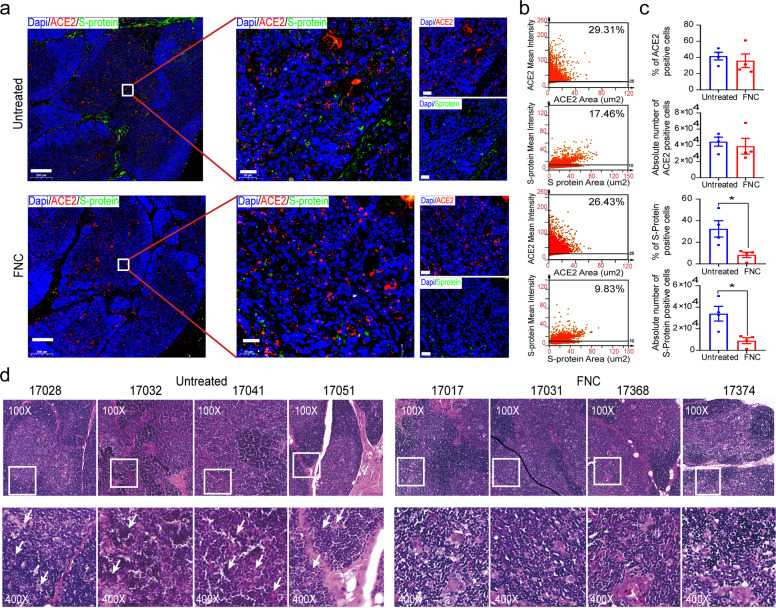
FNC-protected thymus from the damage by SARS-CoV-2 infection. By the end of the experiment, the RM monkeys have practiced euthanasia, and the thymus samples were collected. **a** The representative images of the multi-color immunofluorescent staining were for ACE2 (red) and S protein (green) in the thymus of the RM monkeys that were inoculated with SARS-CoV-2 and treated or untreated with FNC. The regions of interest (ROI) are boxed in white, and their magnified photos are shown in the middle, with a decomposition diagram on the right. Scale bars, 200 µm (left) and 20 µm (middle and right). **b** Representative images of tissue flow cytometry analysis, using Tissue FAXS platform and Tissue Quest software (Tissue Gnostics), showed that S-protein positive cells were decreased by FNC. **c** Statistical results of ACE2 and S-protein positive cells in the thymus of the FNC-treated group and the untreated one (*n* = 4 for each group) showed that FNC significantly reduced the cells infected with SARS-CoV-2. **d** FNC alleviated infiltration, effusion, and structure damage (white arrows) in the thymus of the FNC-treated virus (+) monkeys. The regions of interest (ROI) are boxed in white, and their magnified photos are shown below. Data are presented as mean ± SEM (*n* = 4), *p < 0.05, FNC-treated group vs. untreated group, by Mann–Whitney *U* test

To further verify the antiviral effect of FNC in the thymus, viral S-protein in the thymus was detected with immune staining. The representative staining result is demonstrated in Fig. 5a, shown under low magnification (left), high magnification (middle), and decomposition diagram (right). Tissue ACE2 was stained red, and viral S-protein was stained green. More green signals were detected in the thymus of the untreated monkey than in the FNC-treated one. Viral S-protein was quantitatively measured in the thymus with the Tissue FAXS platform and Tissue Quest software (Tissue Gnostics).^10,26–30^ As shown in Fig. 5b, c, no significant difference was found in the relative proportion and absolute numbers of ACE2^+^ cells between the untreated and FNC-treated monkeys (41.73 ± 9.46% vs. 35.96 ± 16.90%; 44,525 ± 11,059 vs. 38,929 ± 19,470 cells; *n* = 4 for each group). However, the number of S-protein-positive cells in the untreated monkeys was much higher than that in the FNC-treated ones (32.42 ± 15.00% vs. 8.36 ± 4.84%; 33,933 ± 13,538 vs. 8941 ± 5436 cells; *n* = 4 for each group). The results of all eight monkeys are shown in Supplementary Fig. 10. Accordingly, the thymus of the untreated virus (+) monkeys on Day 8 showed infiltration, effusion, and structural damage in HE staining; but these pathological changes were hardly seen in the thymus of the FNC-treated monkeys (Fig. 5d). Further evaluation of thymus cells undergoing programmed death was done with TUNEL staining, followed by quantitation with Tissue FAXS platform and Tissue Quest software (Tissue Gnostics, described in Methods). As shown in Supplementary Fig. 11, the number of apoptotic thymus cells from the untreated virus (+) monkeys was much higher than that from the FNC-treated ones. The results were also additionally supported by the analysis using single-cell sequencing technique (droplet-based scRNA-seq; 10× Genomics, see below), which showed that much fewer cells were in programmed death after FNC treatment (#17368), especially for the thymus CD4+, CD8+, and NKT cells (Supplementary Fig. 11d).

### Immune enhancing effect of FNC in the SARS-CoV2-infected RM monkeys

**Fig. 6:**
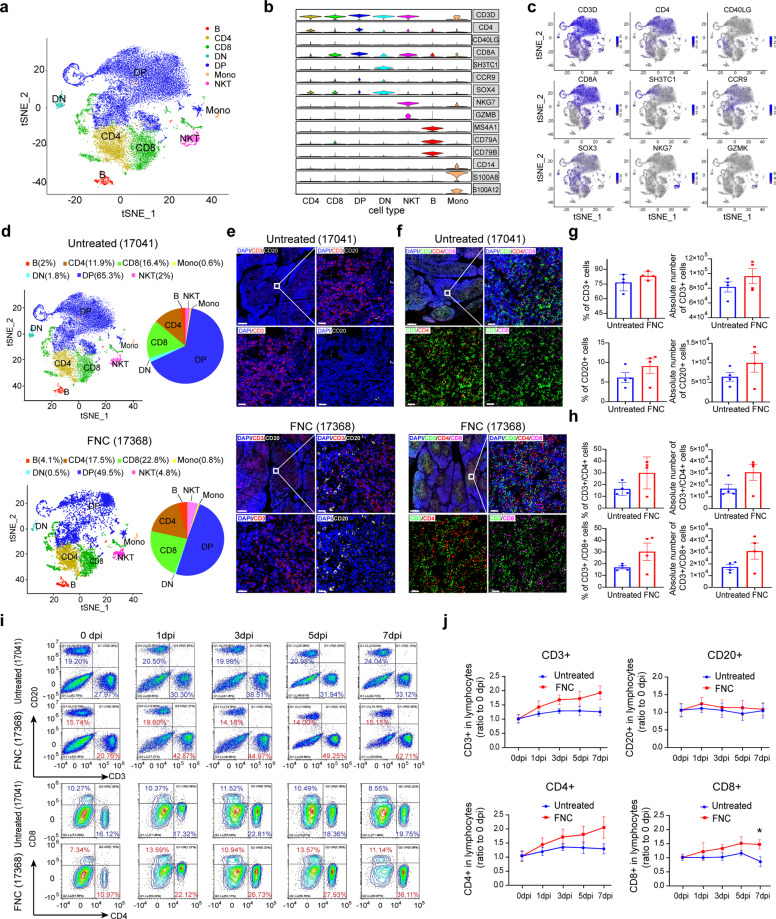
FNC improves the profile of immune cells in RM monkey. The droplet-based scRNA-seq (10Χ Genomics) was performed to characterize the immune cell features in the thymus of RM monkeys inoculated with SARS-CoV-2, untreated (#17041) or treated (#17368) with FNC. **a** Through analysis of 27,751 single cells from the thymus samples of monkey #17,041 (untreated) and #17,368 (treated with FNC), 7 clusters with the respective labels were identified. Each colored dot represents a single cell, according to cell type. **b** Expression of selected canonical cell markers in the 7 clusters showing with violin images. Columns represent clusters and rows represent selected marker genes. **c** The clusters were labeled by canonical cell gene markers (blue dots). **d** Proportion of each cell type from samples of untreated (up) and FNC treated monkey (bottom). **e** The representative images of multi-color immunofluorescent staining were for CD3 (red) and CD20 (white) protein in the thymus from monkey 17,041 (untreated) and 17,368 (FNC treated). The regions of interest (ROI) are boxed in white (up left), and their magnified photos are shown in the upright. The bottom left is for CD3+ cells, and the bottom right is for CD20+ cells. **f** The representative images of multi-color immunofluorescent staining was for CD3 (green), CD4 (red), and CD8 (purple) protein in the thymus of virus (+) monkeys treated or untreated with FNC. The regions of interest (ROI) are boxed in white (up left), and their magnified photos are shown in the upright. Bottom left is for CD3+/CD4+ cells, and the bottom right is for CD3+/CD8+ cells. **g** Relative proportion and absolute numbers of CD3+ cells (up) and CD20+ cells (down) in thymus from the virus (+) monkeys, treated with or without FNC (*n* = 4 for each group). Cells of a given phenotype were identified and quantitated using the Tissue Quest software (Tissue Gnostics). **h** Relative proportion and absolute numbers of CD3+/CD4+ cells (up) and CD3+/CD8+ cells (down) in thymus from virus (+) monkeys, treated with or without FNC. Cells of a given phenotype were also analyzed using the Tissue Quest software (Tissue Gnostics). **i** Representative flow cytometry analysis of CD3+/CD20+ cells (up) and CD4+/CD8+ cells (down) in PBMC samples from #17,041 and #17,368 at indicated time points. **j** FNC increased the % of CD3+, CD4+, and CD8+ cells in PBMC of the virus-infected monkeys, measured with flow cytometer at indicated time points. Data are presented as mean ± SEM (*n* = 4); *p < 0.05, infected monkeys in FNC group vs. those in the untreated group, by Mann–Whitney *U* test. Scale bars for **e** and **f** 200 µm (up left) and 20 µm (upright, bottom left and bottom right)

The droplet-based scRNA-seq (10× Genomics) was done primarily to depict the immunological profile of the thymus in the SARS-CoV-2-infected RM monkeys with or without FNC treatment. Thymus tissues from the viral (+) RM monkeys untreated (coded# 17041) or treated (code# 17368) with FNC were investigated. The two types of monkeys were enrolled in this further analysis mainly because their viral load was similar in the lung and below the detectable line in the thymus. In this COVID-19 monkey model, the viral load in the lung was about 10 times higher than that in the thymus. The analysis of 27,751 single cells from the thymus samples of infected monkeys, treated and untreated with FNC, identified 7 major cell subtypes expressing marker genes (Fig. 6a). These cells were B cells, CD4+ cells, CD8+ T cells, double-negative cells (DN, CD4^−^CD8^−^), double-positive cells (DP, CD4^+^CD8^+^), monocytes, and NK T cells, with their selected canonical cell gene markers shown in Fig. 6b, c.^28^ Of the thymus cells, 20,622 cells were from the untreated viral (+) monkeys, and 7129 were from the FNC-treated viral (+) monkeys. The thymus of the FNC-treated monkeys (code# 17368) showed an increased percentage of alive CD4 (17.5% vs. 11.9%), CD8 (22.8% vs. 16.4%), B (4.1% vs. 2%), and NKT cells (4.6% vs. 2%) compared with that in the untreated viral (+) monkeys (code# 17041), suggesting an improved profile of immune cells in the thymus. In contrast, the monocytes remained stable (Fig. 6d). Then, the representative multi-color immunofluorescence staining was done with antibodies against CD3 (red) and CD20 (white) proteins (Fig. 6e), as well as CD3 (green), CD4 (red), and CD8 (purple) proteins (Fig. 6f) in the thymus from viral (+) monkeys untreated (code# 17041) or treated (code# 17368) with FNC. The image signal of the eight monkeys (*n* = 4 for both groups) was quantitatively analyzed for the positive cells using the Tissue FAXS platform and Tissue Quest software (Tissue Gnostics). As shown in Fig. 6g, h, the thymus of monkeys in the FNC-treated group (*n* = 4 for both groups) showed an increased percentage and absolute number of CD3+, CD20+, CD3+/CD4+, and CD3+/CD8+ cells. The lymphocyte subsets (% and absolute count) in the thymus of the eight individual monkeys are demonstrated in Supplementary Fig. 12 for CD3+ and CD20+ cells and Supplementary Fig. 13 for CD3+/CD4+ and CD3+/CD8+ cells. It should be mentioned here that about 90% of the cells in the monkey’s thymus were T cells in this assay (Fig. 6a, d).

By principle, improved thymus function should promote the immune profile in the peripheral blood. Thus, lymphocyte phenotype was analyzed for blood samples on days 0, 1, 3, 5, and 7 after infection. Oral FNC elevated the percentage of CD3+, CD4+, and CD8+ cells, but not of CD20+ cells in the FNC-treated monkeys (code# 17368), compared with that in the untreated one (code# 17041) (Fig. 6i). The average values and statistics (*n* = 4, for each group) are shown in Fig. 6j; FNC treatment mainly increased the percentage of CD3+, CD4+, and CD8+ cells in the peripheral blood with time, but the percentage of CD20+ cells remained unchanged. The results were, in general, consistent with those in single-cell sequencing.

**Fig. 7:**
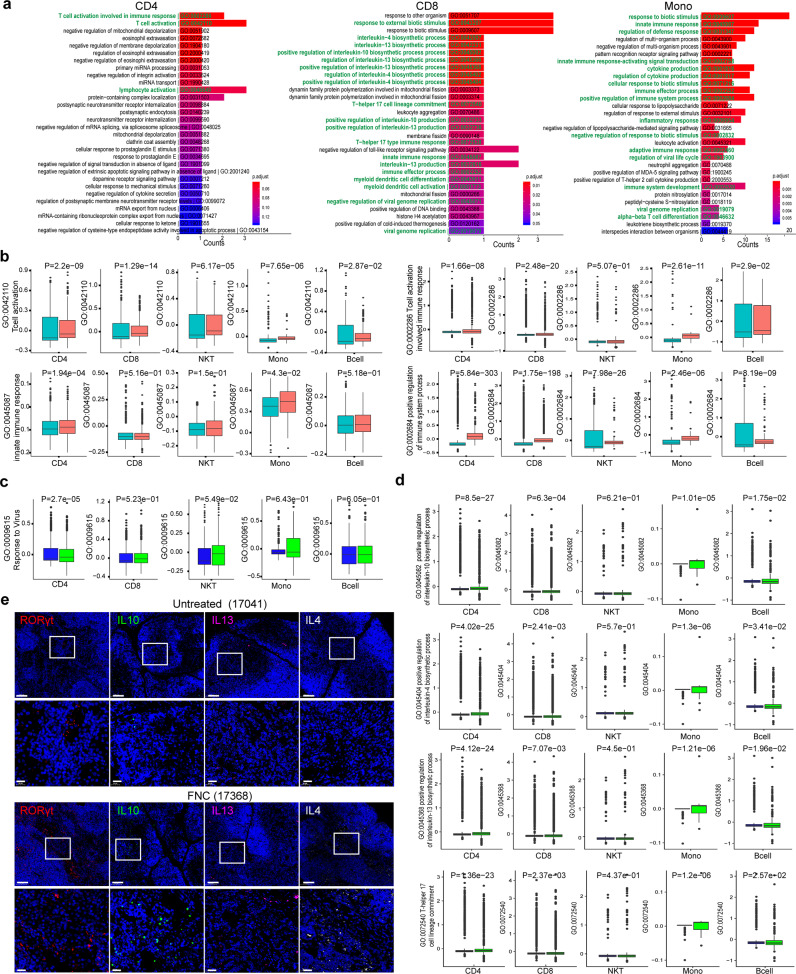
FNC protects the function of the thymus in the virally infected RM monkeys. The droplet-based scRNA-seq (10× Genomics) was performed to characterize the immunological features in the thymus of the SARS-CoV-2(+) RM monkeys, treated or untreated with FNC. **a** The top 30 enriched Gene Ontology (GO) biological process terms in the subset of CD4, CD8, and monocyte are shown. GO terms are labeled with name and ID, and the red-to-blue color indicates the level of *p* values. Interesting terms are labeled in green. **b** Histogram of expression levels of the GO biological process terms, including T-cell activation, T-cell activation involved in immune response, innate immune response, and positive regulation of immune system process, in each subset cells from the infected RM monkeys, treated or untreated with FNC. Horizontal lines represent median values, and the statistical analysis is shown at the top of each figure. Light blue box: untreated (#17041); pink box: FNC treated (#17368). **c** Histogram of expression levels of GO biological process terms related to response to the virus in each cell subset. Horizontal lines represent median values, and the statistical analysis is shown at the top of each figure. Blue box: untreated (#17041); green box: FNC treated (#17368). **d** Histogram of expression levels of GO biological process terms related to IL4, IL10, IL13 biosynthesis, and Th17 lineage commitment in each subset cells. Horizontal lines represent median values, and the statistical analysis is shown at the top of each figure. Blue box: untreated (#17041); green box: FNC treated (#17368). **e** Representative images of immunofluorescent staining of RORγt, IL10, IL13, and IL4 in thymus samples. The regions of interest (ROI) are boxed in white (up), and their magnified photos are shown below. Scale bars, 100 µm (up) and 20 µm (down). *p* Value was analyzed by Mann–Whitney *U* test

Our further study focused on the function of immune cells in the thymus. The gene enrichment analysis of the differentially expressed genes (DEGs) was done in the thymus cells to examine the transcriptomic changes caused by FNC in the thymus of the viral (+) monkeys. We found that the top 30 enriched Gene Ontology (GO) biological process terms were largely associated with DEGs of immunity, antiviral, and inflammatory responses (Fig. 7a and Supplementary Fig. 14). Then, we investigated the expression of nine important pathways through a comparison between FNC-treated (code# 17368) and untreated (code# 17041) viral (+) monkeys. The pathways involved in T-cell activation, T-cell-activation-involving immune response, innate immune response, positive regulation of immune system process (Fig. 7b), response to the virus (Fig. 7c), and IL-4, IL-10, and IL-13 production (Fig. 7d) were analyzed in five major types of cells including CD4+, CD8+, NKT cells, monocytes, and B cells. We found that in all five types of thymus cells, the T-cell activation signal was elevated significantly by FNC, particularly in CD4+ and CD8+ cells. The signal for the T-cell-activation-involving immune process was also elevated, particularly in CD4+ and CD8+ cells and monocytes. The signal for innate immune response in the thymus was significantly promoted by FNC, but mainly in CD4+ cells only. For positive regulation of the immune system process, the signal was largely increased in all five types of cells. The sign for response to the virus after FNC treatment was improved only in CD4+ cells. These results showed the promotion of intracellular pathways for the immune or antiviral response in the major types of cells in the thymus.

Also, the upregulated expression of anti-inflammatory interleukins was found in the thymus of the FNC-treated viral (+) monkeys. As shown in Fig. 7e and Supplementary Fig. 15, the expression of IL-4, IL-13, and IL-10, which were active against IL-6, IL-12, and TNF-α, was elevated in the thymus cells of the FNC -treated viral (+) monkeys (code# 17,368) than those of the untreated ones (code# 17,041). The expression of RORγt related to Th17 cells was also increased by FNC. Immune staining verified that more IL-4, IL-13, IL-10, and RORγt (Fig. 7e and Supplementary Fig. 15) proteins were expressed in the thymus cells of the FNC-treated monkeys, supporting the results obtained from single-cell sequencing (Fig. 7d).

### Treating COVID-19 patients with FNC

FNC on compassionate use in the clinical treatment of COVID-19 was presented below. The clinical study was a randomized, single-arm, and multicenter trial. The primary goal of the trial is to learn whether oral FNC could inhibit the SARS-CoV-2 virus and shorten the disease course. A total of 33 COVID-19 patients positive for SARS-CoV-2 RNA were investigated. Among them, 1 withdrew from the study due to the medical transfer to another hospital, and 1 patient refused to take the chemical drugs after enrollment, leaving 31 subjects in the study. The patients were categorized into two groups: patients with severe diseases (*n* = 5) and those with moderate COVID-19 (*n* = 26), according to their clinical manifestation.^28^ The average age of the 31 patients was 50.19 ± 18.90, with 9 males (age, 48.22 ± 19.57) and 22 females (age, 51.00 ± 19.03). FNC was given orally (started with 10 mg, qd, for the first day, followed by 5 mg, qd, *n* = 8; or started with 5 mg, and remained the same dose for subsequent treatment, *n* = 23). Major clinical manifestation, laboratory tests, chest CT, dates of admission, and discharge from the hospital were evaluated in a comprehensive fashion.

As shown in Table 1, the 31 patients had been treated with traditional Chinese Medicine (TCM) or other antiviral drugs, such as interferon-α, lopinavir/ritonavir-Kaletra, ribavirin, chloroquine, favipiravir, Ganciclovir, Oseltamivir, and arbidol, but the treatment failed; then they entered the FNC study. As shown in Table 1a, the average time from SARS-CoV-2 positive to FNC treatment was 23.65 ± 16.80 days (3–62 days in range). No patients died in the trial, and all patients (31/31, 100%) had their SARS-CoV-2 RNA turned negative and were discharged from the hospital after FNC treatment, with no exception. The average time from FNC treatment to the negative conversion of SARS-CoV-2 RNA was 3.29 ± 2.22 days (range: 1–9 days). The average time from FNC treatment to discharge from the hospital was 9.00 ± 4.93 days (range: 2–25 days). The average FNC treatment time was 6.77 ± 2.74 days (range: 2–12 days). The outcome of each individual patient is shown in Supplementary Tab 3. The results indicated a good response to FNC treatment in the entire study cohort. Of the 31 patients, 11 were first treated with known antiviral drugs before but the treatment failed; then these 11 patients entered the study and were treated with FNC as the only antiviral drug. Thus, a self-controlled comparison was made (after failure with other antiviral treatment, FNC was used in these 11 patients) for these patients and the results are shown in Table 1b. FNC showed a significant therapeutic effect compared with the known antivirals (in previous treatment), with respect to the average time from drug treatment to negative conversion of nucleic acid, time from drug treatment to discharge from the hospital, duration of drug treatment, and recovery rate. Of the 31 patients, 26 were classified as moderate COVID-19 and 5 as severe COVID-19, but the time needed for SARS-CoV-2 RNA negative conversion and discharge from the hospital after FNC treatment appeared not statistically different between the two groups (Table 1c). A comparison between the patients who used FNC only (*n* = 15) and those who took FNC plus TCM or/and other antivirals (interferon-alpha, arbidol, Lopinavir/ritonavir, chloroquine, *n* = 16) showed that FNC alone probably achieved the better therapeutic outcome. The time from FNC treatment to viral negative conversion (2.13 ± 1.30 vs. 4.38 ± 2.39, *p* = 0.0032); the time from FNC treatment to discharge from the hospital (8.67 ± 6.24 vs. 9.31 ± 3.46, *p* = 0.7219) and the average time of FNC treatment (6.20 ± 2.60 vs. 7.31 ± 2.85, *p* = 0.2658) were not statistically different between the two groups (Table 1d). Doubled first dosing of FNC (10 mg, qd,) caused no difference in clinical outcome, as compared to that given the first dose at 5 mg (qd, data not shown). The main side-effect after FNC treatment was transient dizziness and nausea in the early treatment (first 1–2 days) and occurred in 16.12% of the patients (5/31).

**Table 1:** Therapeutic efficacy of FNC in patients with COVID-19. **a** General outcome of the patient cohort after FNC treatment. **b** Outcome comparison: known antivirals vs. FNC (a self-controlled comparison). **c** Outcome comparison after FNC therapy: severe COVID-19 patients vs. those with moderate disease. **d** Outcome comparison after FNC therapy: FNC plus other antivirals vs. FNC alone

a							
*N* = 31	Age	Time from virus-positive to FNC treatment (days)	Time from FNC treatment to SARS-CoV-2 negative conversion (days)	Time from FNC treatment to discharge from the hospital (days)	Duration of FNC treatment (days)	Recovery rate	Side effect
Average	50.19	23.65	3.29	9.00	6.77	100% (31/31)	16.12% (5/31)
STDEV	18.90	16.80	2.22	4.93	2.74		
Median	51.0	24.0	3.0	9.0	7.0		
Range	20–81	3–62	1–9	2–25	2–12		

b					
Treatment	Age	Time from drug treatment to SARS-CoV-2 negative conversion (days)	Time from FNC treatment to discharge from the hospital (days)	Duration of drug treatment (days)	Recovery rate
Drug Rx#, before FNC (*n* = 11)	53.00 ± 19.75	NA*	NA*	16.36 ± 9.91	0 (0/11)
FNC (*n* = 11)	53.00 ± 19.75	1.73 ± 0.79	9.09 ± 7.27	6.00 ± 3.03	100% (11/11)
*p* Value				0.0034^	0.0000£

c							
Severity	Age	Time from virus-positive to FNC treatment (days)	Time from FNC treatment to SARS-CoV-2 negative conversion (days)	Time from FNC treatment to discharge from the hospital (days)	Duration of FNC treatment (days)	Recovery rate	Side effect
Severe *n* = 5	50.60 ± 21.05	21.20 ± 14.65	3.00 ± 2.92	5.60 ± 3.78	5.00 ± 3.32	100% (5/5)	40% (2/5)
Moderate *n* = 26	50.12 ± 18.91	24.12 ± 17.41	3.35 ± 2.13	9.65 ± 4.91	7.12 ± 2.55	100% (26/26)	11.5 (3/26)
*p* Value	0.9592	0.7288	0.7558	0.0922	0.1155		

d							
Drug combination	Age	Time from virus-positive to FNC treatment (days)	Time from FNC treatment to SARS-CoV-2 negative conversion (days)	Time from FNC treatment to discharge from the hospital (days)	Duration of FNC treatment (days)	Recovery rate	Side effect
Yes *N* = 16	47.56 ± 18.30	17.50 ± 11.89	4.38 ± 2.39	9.31 ± 3.46	7.31 ± 2.85	100% (16/16)	18.8% (3/16)
No *N* = 15	53.00 ± 19.75	30.20 ± 19.08	2.13 ± 1.30	8.67 ± 6.24	6.20 ± 2.60	100% (16/16)	13.3% (2/15)
*p* Value	0.4327	0.0330	0.0032	0.7219	0.2658		

^@^Self-controlled comparison: These 11 patients were first treated with known antivirals; after failure in the early treatment, they were treated with FNC alone

^#^Drugs: interferon-alpha, arbidol, Lopinavir/ritonavir, chloroquine, ribavirin, or antiviral TCM, with one or more in combination

*Not applicable (no negative conversion, or no discharge from the hospital, after 4–32 days of treatment)

^£^Chi-square test

^^^Unpaired student’s *t* test

Mean ± SD

*P* value by unpaired *t* test

Mean ± SD

*P* value by unpaired *t* test

## Discussion

The ongoing outbreak of SARS-CoV-2 infection has caused the death of many patients globally. Although several known drugs and laboratory agents have shown anti-SARS-CoV-2 activity, no antiviral agents have been proven to be both highly effective and safe in humans. FNC is an RdRp inhibitor for RNA viruses.^15,17^ The anti-coronavirus activity of FNC might not be as potent as that of remdesivir in the in vitro test; however, its clinical efficacy in curing COVID-19 was significant, showing inhibition of SARS-CoV-2 replication in all 31 patients after 3.29 ± 2.22 days on FNC therapy, consistent with a pilot study published before.^31^ The anti-coronavirus activity of FNC was also evidenced in animal experiments using RM. Interestingly, the dose of FNC in humans was only 5 mg per day (oral), much lower than that of remdesivir (100 mg per day, iv). The chemical analysis of the drug distribution in vivo revealed that FNC and its triphosphate are largely concentrated in the thymus and PBMCs, suggesting an immune-targeting nature of FNC on top of its antiviral effect, different from that of remdesivir and unique in known RdRp inhibitors. This might explain the high efficiency of FNC in treating COVID-19 and suggest the thymus as a key organ in defense against SARS-CoV-2-induced fatal diseases. The monkey experiments showed that the inhibition of SARS-CoV-2 replication in the thymus might protect the host immune system from viral attack and promote host T-cell immunity against viruses. Thus, this mode of action can be viewed as a chemo-to-immune dual-phase antiviral therapy, which may be suitable for viruses that target the immune system such as AIDS and COVID-19. In fact, based on the results of previous clinical studies,^31^ FNC has been in phase III clinical trial in China for COVID-19 and has been approved by China FDA for AIDS treatment on July 21, 2021 (XZXK-2021-214).

In this pandemic, COVID-19 clinical outcomes ranged from asymptomatic to acute respiratory failure and death,^32^ and the decisive factors for this big diversity are of interest. Among the possible factors, the host immunity against SARS-CoV-2 is an epicenter. In the SARS-CoV infection in 2003, CD4+ responses positively correlated with good outcomes,^33^ and recent results demonstrated the possible role of T cells in SARS-CoV-2 infection.^34^ The results from the present study on FNC, which concentrated in the thymus, provided good evidence for the importance of T-cell-mediated anti-SARS-CoV-2 immunity, and further pinpointed that the thymus might be the key organ in the control of COVID-19.

The human thymus in the chest is a primary immune organ and the birthplace of circulating T lymphocytes responsible for the host immunity in general. The aging human thymus shrinks significantly, along with the reduced immunity and distorted immune regulation in older adults. In this SARS-CoV-2 pandemic, one of the important abnormalities in the blood is lymphopenia caused by SARS-CoV-2,^7,9,35^ indicating damage to the immune system. Furthermore, elevated cytokine levels (IL-1β, INF-γ, TNF-α, and IL-6),^19–24,36^ which are also closely related to the function of lymphocytes or monocytes, are critical change in COVID-19 and can cause severe cytokine storm and death.^9^ Damaged regulatory function in T cells can be an important factor responsible for the abnormal production of cytokines. Indeed, aged patients with COVID-19 have much higher mortality compared with those in middle or young age,^5,37^ probably because their defensive immunity and regulatory function in lymphocytes or monocytes are injured. FNC treatment in viral (+) monkeys increased the levels of IL-4, IL-10, and IL-13 in the thymus, but not of IL-1β, INF-γ, TNF-α, and IL-6, suggesting a biological response against cytokine storm.^38–45^ Thus, we assume that the high efficiency of FNC in treating COVID-19 may be mediated via at least two steps: antiviral action in the thymus and subsequent promotion of immunity against viral infection for the entire body. How the thymus has most of the active forms of FNC is not known. However, this organ and the chemo-to-immune antiviral mode of action can be a rational strategy for designing drugs against SARS-CoV-2.

The clinical results showed that FNC cured the COVID-19 disease in almost all the patients. Patients with severe COVID-19 showed a good response to the drug-like those diagnosed with moderate COVID-19. The therapeutic efficacy of FNC seemed to be better than that reported for remdesivir,^46,47^ and its side effects were mild and transient, less severe than those of remdesivir.^46,47^ However, the conclusive efficacy evaluation of FNC requires further randomized, placebo-controlled, and large-scale clinical trials in the future.

The original plan for this clinical study was to enroll 80 patients with COVID-19 for FNC treatment in the 3 hospitals. However, only 33 entered the trial because the number of patients with COVID-19 decreased quickly after successful control of the pandemic in February and March 2020 in China. Furthermore, the SARS-CoV-2 viral ribonucleic acid in swab samples was qualitatively detected in local CDC laboratories, not quantitatively, because this was the only type of SARS-CoV-2 assay kit approved for clinical use by the China FDA at that time. Therefore, all the patients were considered positive or negative for SARS-CoV-2 after at least two consecutive tests to ensure the viral test results. In addition, chest CT was another clinical indication to confirm the diagnosis. Also, as this urgent FNC clinical study was done in the early outbreak of COVID-19 in February–March 2020, the flow cytometric analysis of lymphocyte subsets for patients with COVID-19 was not available in hospitals at that time. Therefore, we used the monkey model to examine the effect of FNC on the immune system. The lack of a randomized control group was another restriction for the interpretation of results. These limitations of the clinical study should be overcome in the Phase III clinical trial of FNC, in which the quantitative assay of SARS-CoV-2 ribonucleic acid and lymphocyte phenotyping have been included.

Monkeys infected with SARS-CoV-2 were used in the investigation to confirm the therapeutic efficacy of FNC in vivo. We did observe a significant therapeutic effect of FNC on the inhibition of viral load, promotion of lymphocyte subsets, protection of histological structures, reduction of SARS-CoV-2-caused inflammation, and improved chest x-ray image. Although a significant reduction in viral load by FNC was found in the nasal swabs and blood in the viral copy measurement, viral titration assay might provide additional information because it measures the number of live viruses.^48^ The negative detection for the viral nucleic acids in the thymus of the FNC-treated monkeys (on Day 8) was consistent with the thymus-homing feature of FNC. Accordingly, the histological structure of the thymus of monkeys was well protected by FNC from virus-caused damage, which might subsequently contribute to the improved profiles of the lymphocyte subsets seen in the FNC-treated monkeys. We assumed that the improved lymphocyte profile by FNC could be very important for the final clearance of SARS-CoV-2 in the body. Currently, the role of T cells in COVID-19 is the focus of immunological research;^49^ the results of the present study might be informative. However, this monkey model might be considered a case of moderate but not severe COVID-19, as the bodyweight change was irregular in the infection course and cough and rhinorrhea were not seen. Also, body temperature changed erratically. We consider that the monkeys at 3–4 years of age might be too young to get severe COVID-19, and the viral infection dose (1 × 10^6^ pfu) might be lower than that required for disease development. Despite limitations, this monkey model experiment provided solid evidence for FNC with respect to its homing to the thymus, inhibitory effect on SARS-CoV-2, promotion of immune response, reduction of lung tissue damage, and therapeutic efficacy in vivo. As only four monkeys were included in each study group, the statistical significance was highly valuable.

In the last 20 years, SARS in 2003, MERS in 2012, and the current pandemic of COVID-19 have provoked strong attention worldwide. It is foreseen that coronavirus infection via cross-species transmission can be a longstanding threat to public health in the years ahead. Here, we seem to identify FNC as a highly effective drug against SARS-CoV-2 and the thymus as a key organ for anti-COVID-19 immunity. We consider the discovery of FNC very crucial to cope with the current COVID-19 and future epidemics of coronavirus.

### Statistical analysis

All data were analyzed with GraphPad Prism 8.0 software (GraphPad, San Diego, CA). Statistically significant differences were determined using unpaired Student’s *t* test and Mann–Whitney *U* test according to experiment requirements. *p* Value < 0.05 was considered statistically significant, *p < 0.05, **p < 0.01, ***p < 0.001. No statistical methods were used to predetermine sample size.

## Supplementary information

Supplementary materials

## Data Availability

All raw data are available from the corresponding author on reasonable request.
