# Supplementary material for: Azvudine is a thymus-homing anti-SARS-CoV-2 drug effective in treating COVID-19 patients
Source: Signal Transduct Target Ther. 2021 Dec 6;6:414. doi: 10.1038/s41392-021-00835-6 (PMC8646019; doi:10.1038/s41392-021-00835-6)
Supplement: Supplementary file 1 — Supplementary materials [file 41392_2021_835_MOESM1_ESM.docx]

Supplementary Materials for

Azvudine Is a Thymus-Homing Anti-SARS-CoV-2 Drug Effective in Treating COVID-19 Patients

Jin-Lan Zhang^1,#^, Yu-Huan Li^2,#^, Lu-Lu Wang^1,2,#^, Hong-Qi Liu^3,#^, Shuai-Yao Lu^3,#^, Yong Liu^4^, Ke Li^2^, Bin Liu^5^, Su-Yun Li^6^, Feng-Min Shao^7^, Kun Wang^2^, Ning Sheng^1^, Rui Li^1^, Jin-Jin Cui^2^, Pei-Chun Sun^7^, Chun-Xia Ma^3^, Bo Zhu^8^, Zhe Wang^1^, Yuan-Hao Wan^4^, Shi-Shan Yu^1^, Yongsheng Che^2^, Chao-Yang Wang^4^, Chen Wang^9^, Qiangqian Zhang^10^, Li-Min Zhao^1^, Xiao-Zhong Peng^3,^* , Zhen-Shun Cheng^5,^*, Jun-Biao Chang^8,10^*, Jian-Dong Jiang^1,2,^*

Correspondence to: jiang.jdong@163.com, cjb@htu.edu.cn, [pengxiaozhong@pumc.edu.cn](mailto:pengxiaozhong@pumc.edu.cn), cheng_zhenshun@126.com

**Materials and Methods**

Anti-SARS-CoV-2 assay *in vitro*

*Cells, virus, and antivirals*

Vero E6 cells and Calu-3 cells were obtained from Cell Center, Institute of Basic Medical Sciences. Vero E6 cells were maintained in Dulbecco’s modified Eagle’s medium (DMEM; Gibco Invitrogen) supplemented with 10% fetal bovine serum (FBS; Gibco Invitrogen) at 37°C in a humidified atmosphere of 5% CO2. Calu-3 cells were cultured in minimum Eagle’s medium (MEM; Gibco Invitrogen) supplemented with 10% FBS at 37°C with 5% CO2 atmosphere. The clinical isolate COVID-19 virus was obtained from the Center of Diseases Control, Guangdong Province China, as described before. All the live COVID-19-related experiments were performed in a biosafety level-3 (BLS-3) laboratory.

*Evaluation of anti- SARS-CoV-2 activities of the CL-236*

The cytotoxicity of the tested drugs in Vero E6 or Calu-3 Cells were determined by Cell Titer 96® Aqueous one Solution Cell Proliferation Assay (Promega, US). To evaluate the antiviral efficacy of the drugs, Vero E6 or Calu-3 cells were cultured overnight in 96-well plate with a density of 4 ×10^4^ cells/well. Cells were pre-treated with the different doses of the indicated antivirals for 1 h. Viruses (MOI=0.05) were subsequently added to cells, followed by 1 h incubation. Then, the virus-drug mixture was removed and cells were further cultured with fresh drug-containing medium. At 48 h post infection (pi), the culture supernatant was collected and lysed in Trizol (Thermo, Cat no. 15596018) for further analysis. Remdesivir was tested as reference.

*Viral RNA extraction and quantitative RT-PCR (qRT-PCR)*

150 μl of cell culture supernatant was harvested for viral RNA extraction using the Direct-zol RNA miniprep Extraction Kit (Zymo Research, Cat no R2052) according to the manufacturer’s instructions. 50 μl of DNase/RNase-Free Water was used to elute RNA. Quantitative RT-PCR was used to quantify viral genome in samples using TaqMan Fast Virus 1-Step Master Mix (ThermoFisher, US) with purified viral RNA of SARS-CoV-2 as a standard curve. RT-PCR was performed on CFX384 Touch Real-Time PCR Detection System (BioRad, US). Conditions for RT-PCR were used as follows: 25°C for 2min, 50°C for 15min, 95°C for 2min, then 40 cycles at 95°C 5sec and 60°C 31sec. Primers and probe, specific for NP gene was synthesized according to sequences reported by China CDC, Target-2-F：GGGGAACTTCTCCTGCTAGAAT, Target-2-R：CAGACATTTTGCTCTCAAGCTG, Target-2-P：5'-FAMTTGCTGCTG CTTGACAGATTTAMRA-3'. Data was plotted via the software GraphPad.

Anti- HCoV-OC43 assay *in vitro*

*Cells, virus, and antivirals*

Human lung cancer (H460) cells were were kindly provided by Dr. Zhen Wang, at Institute of Medicinal Biotechnology, Chinese Academy of Medical Sciences and Peking Union Medical College and cultured in Dulbecco’s Modified Eagle Medium (Invitrogen, Carlsbad, CA, USA) supplemented with 10% FBS (Invitrogen, Carlsbad, CA, USA) and antibiotics (100 U/ml penicillin and 100 mg/ml streptomycin) (Invitrogen, Carlsbad, CA, USA) at 37°C in a 5% CO2 incubator. HCoV-OC43 (strain VR1558) was a kind gift from Dr. Xuesen Zhao, Beijing Ditan Hospital, Capital Medical University and was amplified in HCT-8 cells according to the instruction from ATCC. CL-236 with purity over 98% was provided by Dr. Jun-Biao Chang of the Henan Normal University. Ribavirin (RBV) was from Sigma–Aldrich (St. Louis, MO, USA). CL-236 (10 mg/ml) was dissolved in DMSO and RBV (10 mg/ml) was dissolved in the culture medium. The study drugs were diluted with maintenance medium (2% FBS) to a final working concentration in the experiments.

*Anti- HCoV-OC43 experiment*

For the antiviral test against HCoV-OC43, H460 cells were plated into 96-well or 12-well culture plates and infected with HCoV-OC43 (MOI=0.05). Then, various concentrations of CL-236 were added at the same time for 48 h incubation and then the N protein was analyzed using immunofluorescence analysis or determined by Western blot.

*Western blot analysis*

Western blot analysis for the N protein was done as following. H460 cells were lysed in the M-PER mammalian protein extraction reagent (Thermo, Rockford, IL, USA) containing halt protease inhibitor single-use cocktail (Thermo). About 10 μg proteins were denatured and applied to sodium dodecyl sulfate-polyacrylamide gel electrophoresis (SDS-PAGE). The electrophoresis products were transferred to a polyvinyl idenefluoride (PVDF) film and PVDF membranes were then incubated at room temperature with specific primary antibody. After a standard washing, membranes were incubated with horse radish peroxidase (HRP)-labeled secondary antibody. The signal was developed using a chemiluminescent substrate. The primary antibodies used in this study included antibodies against β-actin (Cell Signaling Technology, Boston, MA, USA) and Coronavirus N protein (Millipore, Bedford, MA, USA). The goat anti-rabbit and anti-mouse HRP-labeled antibodies were obtained from Cell Signaling Technology.

*Immunofluorescence staining analysis*

Immunofluorescence staining was also used to see N protein expression. The H460 cells (1.5 × 10^4^ cells/well) seeded into 96-well were infected with HCoV-OC43 (MOI=0.05), and CL-236 were added at the same time followed by 48 h incubation. The culture medium then was removed and the cells were washed and fixed. The cells were permeabilized in 0.5% Triton X-100 at room temperature for 15 min and blocked in PBS containing 1% BSA for 60 min at room temperature. Cells were then incubated with an anti-coronavirus N antibody (Millipore) at a dilution of 1: 200 for 2 h at room temperature. After washing 3 times with PBS, the samples were reacted with Alexa Fluor 488-labeled goat anti-mouse secondary antibody (Beyotime Institute of Biotechnology, China) for 1 h at room temperature. After washing, images were taken using a fluorescence microscope (X-Cite 120；Zeiss Carl Zeiss Meditec, Oberkochen, Germany).

Chemical analysis for FNC

*Chemicals and reagents*

FNC and its tri-phosphorylated metabolite (FNC-TP) standards (with purity over 98%) were supplied by Prof. Junbiao Chang of Henan Normal University (Xinxiang, Henan). Lamivudine (3TC, with 98% purity) as internal standard was supplied by National Institutes for Food and Drug Control (Beijing, China). Succinate-d4 as internal standard was purchased from Cambridge Isotope, Inc. (Woburn, MA, USA). LC-MS grade methanol and acetonitrile were bought from Mallinckrodt Baker Inc. (Phillipsburg, NJ, USA). Ammonium acetate and ammonium hydroxide were obtained from Sigma-Aldrich (St. Louis, MO, USA). Ultra-pure water was prepared using a Milli-Q purification system (Millipore, Bedford, MA, USA). Phosphate buffered saline was purchased from Gibco (Thermo Fisher Scientific, USA). Histopaque-1083 was purchased from Sigma-Aldrich (St. Louis, MO, USA). Protease inhibitor cocktail was purchased from Gene-Protein Link Biotech, China (Lot. P10C01). Phosphatase inhibitor cocktail was purchased from Gene-Protein Link Biotech, China (Lot. P10C03 and P10C04)

*Biodistribution in rats*

25 male Sprague-Dawley rats (200 ± 15 g) were purchased from Vital River Laboratories Co., LTD (Beijng, China). The animals were housed under specific pathogen-free conditions (12 h light/12 h dark photoperiod, 25 ± 2°C, 50±5% relative humidity). All rats were allowed to acclimate for 1 week before experiments. Research was conducted in accordance with all institutional guidelines and ethics and approved by the Laboratories Institutional Animal Care and Use Committee of the Chinese Academy of Medical Sciences and Peking Union Medical College.

Rats were divided randomly into five groups with five rats in each, and were administered with azvudine at a single dose of 5 mg/kg. At the time point of 1h, 2h, 6h, 12h and 24h after drug administration, rats in one group were dissected with euthanization and blood samples were collected using heparin as an anticoagulant. 0.5 ml of blood was transferred to another 1.5ml eppendorf tube to obtain plasma by centrifugation (3500 r/min ×15 min). The tissues, including heart, liver, spleen, lung, kidney, brain, thymus, testis, and epididymis, were collected, and washed with cold 0.9% normal saline, and then kept at -80°C until analysis.

Peripheral blood mononuclear cells (PBMCs) were isolated from blood using Histopaque-1083 according to the manufacturer’s protocol. Firstly, 2 mL of blood collected in heparin sodium tube was diluted with 2 mL of PBS solution. Then, 4mL of diluted blood was layered on top of 4 mL Histopaque-1083 and centrifuged for 30 min at 2000 r/min. PBMCs were then aspirated, washed twice with PBS solution, suspended in PBS, and counted with a hemocytometer. Furtherly, PBMCs were centrifuged (2000 r/min ×10 min), lysed with 1ml of cold 80% methanol for 12h, then ultrasonic for cell disruption (3min). Finally, the cell lysates were stored in -80°C until sample preparation.

*UHPLC-MS/MS analysis*

Tissue samples were weighed and 2-fold volume of 0.9% saline containing phosphatase inhibitor was added to prepare the homogenate on the ice. Then tissue homogenate was ultrasonic extracted for 5 min in the ice bath. 200μL of plasma or tissue homogenate was transferred into a 1.5 ml eppendorf tube and added 600 μL of methanol solution with internal standards. The sample was vortexed for 5 min and kept at 4°C for 15min, then centrifuged at 13000r/min for 10 min (4°C). Protein precipitation was removed and the supernatant was transferred into another 1.5 mL eppendorf tube and dried under a gentle nitrogen stream. The residue was re-dissolved in 100 μL acetonitrile/methanol (75:25, v/v) for analysis.

The UHPLC–MS/MS analysis was performed on a UHPLC system (1290 series, Agilent Technologies, US) coupled to a triple quadrupole mass spectrometer (Agilent 6470 QQQ). The chromatographic separation was carried out on a Waters ACQUITY UPLC BEH Amide column (2.1×150mm，1.7μm). Mobile phase A was comprised of 10 mmol/L ammonium acetate in water containing 0.5 % ammonium hydroxide. Mobile phase B was comprised acetonitrile/water (90:10, v/v) containing 10 mmol/L ammonium acetate and 0.5 % ammonium hydroxide. A gradient elution was employed using the following program: 0-3 min, 100-65% B; 3-7 min, 65-60% B; 7-8min, 60-56% B. The flow rate was set at 0.3 mL/min, the column oven temperature at 30°C and an injection volume of 5μL. The autosampler was set at 4°C. AJS ESI source conditions were set as followings: sheath gas temperature, 250°C; drying gas temperature, 300°C; sheath gas flow rate, 11 L/min; drying gas flow rate, 5 L/min; capillary voltage, 3500 V; nozzle voltage, 500 V and nebulizer pressure, 45 psi. Multiple reacting monitoring (MRM) mode was performed using the characteristic precursor to product ion transitions as shown in the Table S4a. Analytes were quantified by standard curve samples. The standard curves of blank kidney tissue matrix, plasma matrix and PBMCs matrix were applied for quantitative analysis of azvudine and its metabolites. FNC, FNC-NH+O and FNC-NH+O+CH2 were quantified using FNC standard curves, and the phosphate metabolites of FNC (FNC-MP, FNC-DP, and FNC-TP) were quantified using FNC-TP standard curves.

Chemical analysis for remdexivir

*Chemicals and reagents*

Remdesivir (GS-5734) and nucleoside metabolite (GS-441524, Nuc) were purchased from Shanghai Hanxiang Biological Technology Co., Ltd. (Shanghai, China). Nuc-MP and Nuc-TP was purchased from Shanghai Chenxi Biotechnology Co., Ltd. (Shanghai, China). Other reagents were the same as chemical analysis for azvudine.

*Biodistribution in rats*

10 male Sprague-Dawley rats (250 ± 25 g) were purchased from Vital River Laboratories Co., LTD (Beijng, China). The rats were divided randomly into two groups with five rats in each, and were administered with GS-5734 at a single dose of 100 mg/kg, i.p. At the time point of 2h and 6h after drug administration, rats in one group were dissected with euthanization and blood samples as well as heart, liver, spleen, lung, kidney, brain, thymus, testis, and epididymis were collected for UHPLC-MS/MS analysis as mentioned above.

*UHPLC-MS/MS analysis*

The analysis method was like that for FNC expect that the flow rate was set at 0.4 mL/min and the gradient elution was employed using the following program: 0-1.5min, 100-65%B; 1.5-4min, 65-60%B; 4-5min, 60-56%B. Multiple-reaction monitoring (MRM) mode was performed using the characteristic precursor to product ion transitions (Supplementary Table 4b).

The standard curves of blank kidney tissue matrix, plasma matrix and PBMCs matrix were applied for quantitative analysis of GS-5734 and its metabolites. GS-5734, Nuc, Nuc-MP and Nuc-TP were quantified using their own standard curves, and the metabolites of GS-5734 (Ala-Nuc and Nuc-DP) were quantified using Nuc-MP standard curves.

Anti-SARS-Cov-2 effect of FNC in rhesus macaques

The monkey experiment was approved by the Institutional Animal Care and Use Committee of the Institute of Medical Biology, Chinese Academy of Medical Science (Ethics number: DWSP202006 001). The viral infected animal experiment was carried out in the ABSL-4 Unit of the Kunming National High-level Biosafety Primate Research Center (Kunming, China).

The rhesus macaques’ experiment was done by following the method described before^1,2^. Briefly, healthy rhesus macaques (4 male and 4 female, 3-4 years old) were used for the study. SARS-CoV-2 was from the Center of Diseases Control, Guangdong, China, and amplified in the Vero-E6 cells. Animal groups and experimental schedules were outlined in Fig S4. We inoculated rhesus macaques with 10^6^ pfu/ml SARS-CoV-2 intratracheally (0.5ml) and intranasally (0.5ml). FNC (0.07mg/kg, qd, orally) was given 12 h after viral inoculation and continued for 7 days. The animals were monitored daily for physical examination. Chest X-ray radiograph of the anaesthetized monkeys was done with 55-75v and 8-12.5mA with the Mobile digital medical X-ray photography system (MobileCooper, Browiner China).

Quantification of viral genome in swabs and tissue samples was done as described before^1^. Viral genome copy in samples were quantified with real time RT-PCR, using TaqMan Fast Virus 1-Step Master Mix (ThermoFisher, US) and conducted on the CFX384 Touch Real-Time PCR Detection System (Biorad, US). Purified RNA of SARS-CoV-2 served as a standard curve. The operation conditions of RT-PCR were set as following: 25℃ for 2min, 50℃ for 15min, 95℃ for 2min, then 40 cycles at 95℃ 5sec and 60℃ 31sec. Primers/probe specific for the NP gene were synthesized based on sequences from China CDC: Target-2-F：GGGGAA CTTCTCCTGCTAGAAT,

Target-2-R: CAGACATTTTGCTCTCAAGCTG, Target-2-P：5'-FAM-TTGCTGCTGCTTGACAGATTTAMRA-3'

By the end of the experiment, major organs of the monkeys were taken and checked with naked eye examination. Tests for blood biochemistry and hematology indexes, including lymphocyte analysis and organ function assays, were done based on previous reports^1,2^. The paraffin-bedded tissue sections were prepared for haematoxylin and eosin (HE) staining with standard procedure. The HE slides were captured, followed by pathologist evaluation.

Droplet-based single-cell sequencing

Thymus samples were obtained from rhesus macaques untreated (code# 17041) and treated with FNC (code# 17368). All tissues were processed immediately after isolation using consistent protocols with variation in enzymatic digestion strength. Tissues were digested with collagenase type IV (1.6 mg/ml, Roche) and DNase I (Roche) in RPMI + 10% FBS for 1 h at 37℃ with intermittent shaking. The tissue dissociation was passed through 100 um filter and collected cells by centrifugation at 300 g. The cells were rinsed with RPMI + 10% FBS and resuspended in PBS containing 0.04% BSA.

With single cell 3’ Library and Gel Bead Kit V3 (10x Genomics, 1000075) and Chromium Single Cell B Chip Kit (10x Genomics, 1000074), the cell suspension (300-600 living cells per ml determined by Count Star) was loaded onto a Chromium single cell controller (10X Genomics) to generate single-cell gel beads in the emulsion (GEMs) according to the manufacturer’s protocol. Captured cells were lysed and the released RNA were barcoded through reverse transcription in individual GEMs. Reverse transcription was performed on a S1000TM Touch Thermal Cycler (Bio Rad) with the condition of 53°C for 45 min, followed by 85°C for 5 min, and hold at 4°C. The complementary DNA was generated and amplified, the quality of cDNA was afterwards assessed by an Agilent 4200 (performed by CapitalBio Technology, Beijing). According to the manufacture’s introduction, Single-cell RNA-seq libraries were constructed using Single Cell 3’ Library and Gel Bead Kit V3. The libraries were finally sequenced using an Illumina Novaseq6000 sequencer with a paired-end 150 bp (PE150) reading strategy (performed by CapitalBio Technology, Beijing).

*Single cell RNA-seq data processing*

Raw gene expression matrices were generated for each sample by the Cell Ranger software. The output filtered gene expression matrices were read and analyzed by R software (v.3.6.2) with a R package of Seurat (v.3.0.0). In short, cells whose gene number was less than 200, or gene number ranked in the top 1%, or mitochondrial gene ratio was more than 25% were regarded as abnormal low-quality cells and filtered out. With the function of Normalize Data, the gene expression matrices were normalized and 2,000 features of high cell-to-cell variation were calculated by Find Variable Features function. Using Run PCA function to performed linear dimensionality reduction. With the functions of Elbow Plot, Dim Heatmap and Jack Straw Plot, the true dimensionality was identified. Finally, cells were clustered by the Find Neighbors and Find Clusters functions and executed nonlinear dimensional reduction with the Run TSNE function and then visualization was realized by TSNE.

*Cell-type annotation and cluster marker identification*

After nonlinear dimensional reduction by TSNE, cells were clustered together with common features. The markers of each cluster were found by the function of Find All Markers in Seurat. Clusters were classified and annotated according to the markers of cell types.

*Enrichment analysis*

For GO enrichment, KEGG enrichment, and Reactome enrichment, the analysis of cluster markers using Cluster Profiler R package with Benjamini-Hochberg multiple testing adjustment, using top 20 markers gene of cluster. The results were visualized using R package.

Multi-color immune fluorescence staining

Tyramide Signal Amplification (TSA)^3^ method was used for single staining of IL-4, IL-10, IL13, RORγt and tunnel; double staining of CD3 and CD20, ACE2 and S-protein; as well as triple staining of CD3, CD4 and CD8; ACE2, S-protein and N-protein. In brief, for each marker, the samples were treated with rabbit primary antibody and then incubated with HRP-conjugated goat anti-rabbit secondary antibody. After washing, slides were incubated with fluorescent reagent then heated with microwave oven to remove the combined antibodies. The antibodies were in Table S5.

Microscopy and Quantitative Image Analysis

As described by Kaneko and colleagues,^4^ microscopy-based multi color tissue cytometry was applied for quantitative analysis. In brief, images of the tissue specimens were acquired through the Tissue FAXS platform (Tissue Gnostics).^5,6^ The area of the detected tissue was acquired as a digital gray scale image in four channels with filter settings for FITC, Cy3, Cy5 and DAPI. Cells of each phenotype were identified and quantitated, relative to the positive controls.

Flow cytometric analysis

The PBMC cells were subjected to staining with a panel of immune cell markers. The mAbs used for flow cytometry were recorded in Supplementary Table 5.

Clinical trial

This anti-SARS-CoV-2 clinical study of FNC was conducted in 3 academic hospitals, coordinated by the Institute of Materia Medica, Chinese Academy of Medical Sciences / Peking Union Medical College, and the Henan Normal University. 33 patients with laboratory-confirmed coronavirus disease 2019 (COVID-19) were enrolled in the wards of Zhongnan Hospital of Wuhan University Medical College (n=16), Henan Provincial Peoples Hospital (n=8), the First Affiliated Hospital of Henan University of Chinese Medicine (n=9) between Feb 16, 2020 and Mar 20, 2020. One patient enrolled at the Zhongnan Hospital was transferred to another hospital and one patient refused to take any chemical drug after enrollment at the Zhongnan Hospital, leaving 31 subjects in the study. All patients diagnosed COVID-19 with positive reading for SARS-CoV-2 by polymerase chain reaction (PCR), fever and abnormal findings on chest CT were eligible for the study inclusion. FNC was given orally at 10 mg (qd) for the first day and 5 mg (qd) in the subsequent treatment (n=8), or 5 mg (qd) for the entire treatment (n=23). Of the 31 patients, 16 were treated with FNC only, and other 15 treated with FNC plus traditional Chinese medicine or/and known antivirals (interferon-alpha, arbidol, chloroquine). Data were collected by well-trained doctors and documented using standardized report forms. Major clinical manifestation, laboratory tests, chest CT, dates of admission and discharge from the hospital were recorded. The negative conversion rate of SARS-CoV-2 viral nucleic acid was used as the primary endpoint (negative results for at least successively twice), and clinical improvement (discharge from the hospital) was the secondary endpoint.

The clinical study was a randomized, single-arm, and multicenter trial; the primary goal of the trial is to see whether FNC could inhibit SARS-CoV-2 virus and shorten the COVID-19 course. The clinical studies were approved by the University Ethics Committees and their international clinical trial registration numbers are ChiCTR2000030041, ChiCTR2000030424 and ChiCTR2000030487, respectively for the 3 hospitals mentioned above. Informed consents were obtained from the participants before the study.

*SARS-CoV-2 viral RNA test for clinical samples*

SARS-Co-2 viral RNA was detected at the local CDC-laboratories, using RT-PCR. Patient RNA samples of the upper (nasopharyngeal or oropharyngeal swabs) and lower respiratory tract specimens were extracted with the RNA extraction kit 20170583 from DAAN Gene (Guangzhou, China) and qualitatively analyzed with the SARS-CoV-2 RNA PCR detection kit (DAAN Gene), using ABI Prism7500 for reaction and analysis. The baseline detection covers ORF1ab gene and N-gene.

Reference

1. Lu, S., Zhao, Y., Yu, W., Yang, Y. & Peng, X. Comparison of SARS-CoV-2 infections among 3 species of non-human primates. (2020).

2. Rahi, M. S. *et al.* Hematologic disorders associated with COVID-19: a review. *Ann Hematol*. **100**, 309-320 (2021).

3. Toda Y Fau - Kono, K. et al*.* Application of tyramide signal amplification system to immunohistochemistry: a potent method to localize antigens that are not detectable by ordinary method. *Pathology International* **49** (2010).

4. Kaneko, N. et al*.* Loss of Bcl-6-Expressing T Follicular Helper Cells and Germinal Centers in COVID-19. *Cell*. **183**, 143-157.e113 (2020).

5. Zhang, J.-Y. et al*.* Single-cell landscape of immunological responses in patients with COVID-19. *Nature Immunology*. **21**, 1107-1118 (2020).

6. Park, J.-E. et al*.* A cell atlas of human thymic development defines T cell repertoire formation. *Science*. **367**, eaay3224 (2020).


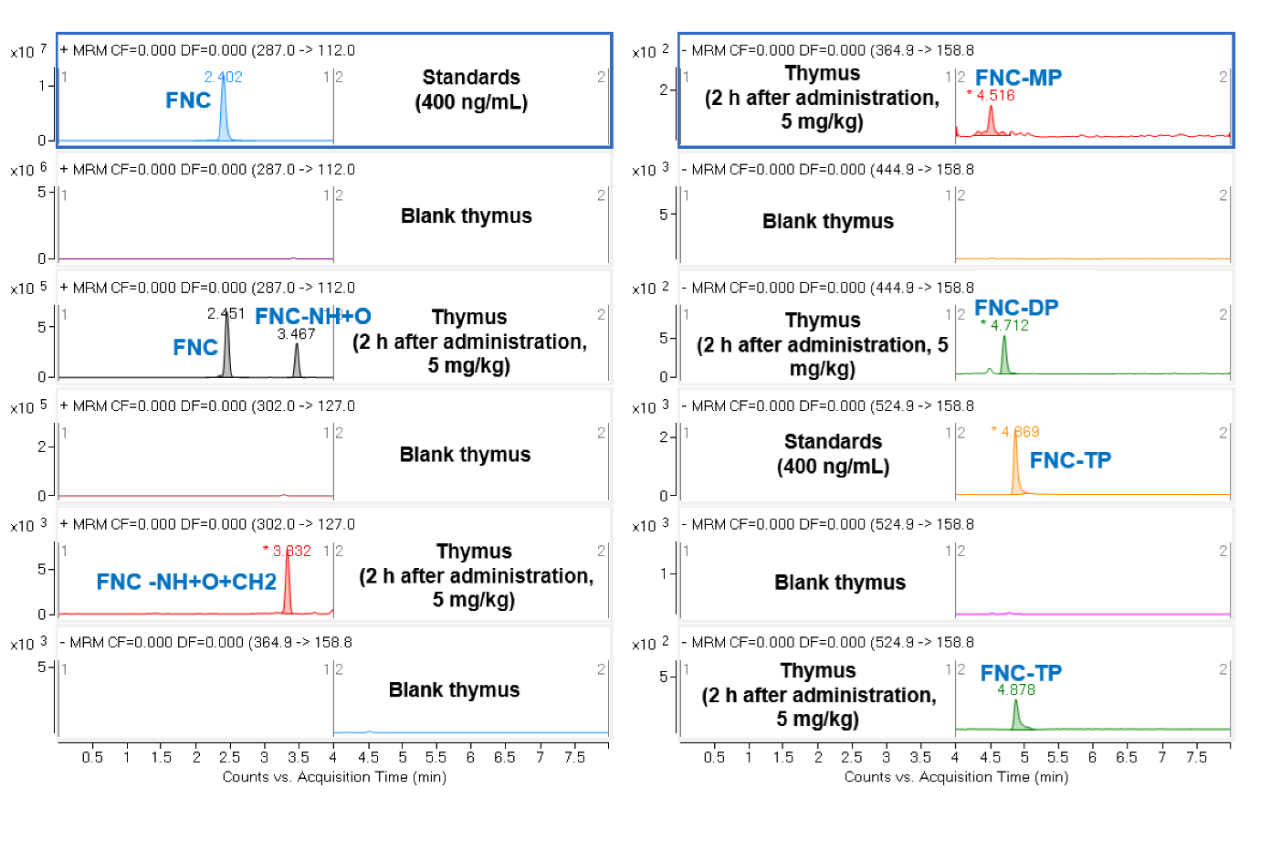
 **Figure. S1. Typical UHPLC-MS/MS chromatograms of FNC and its metabolites in rat thymus and standard solution.**


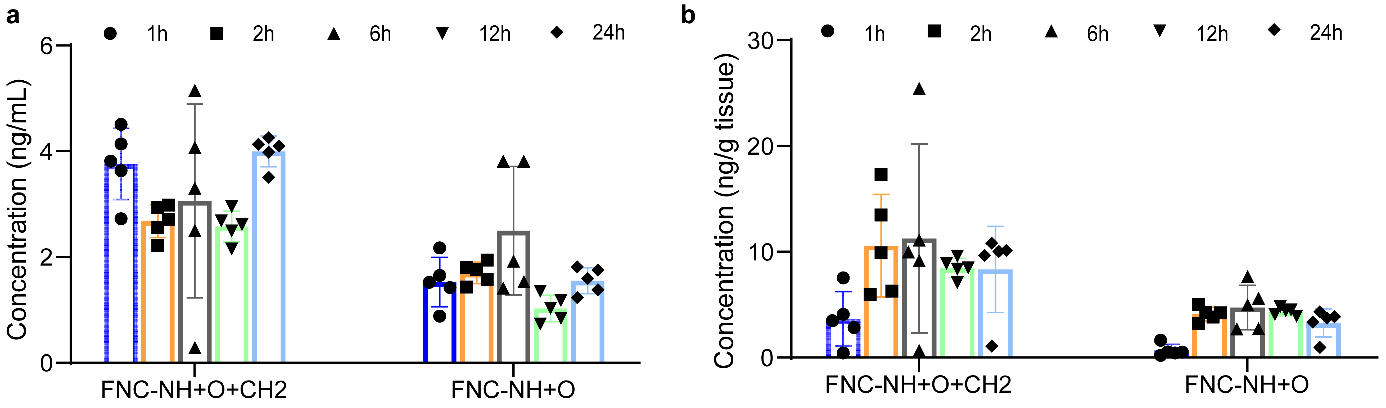
 **Figure. S2. Concentration of FNC metabolites in plasma and thymus.**


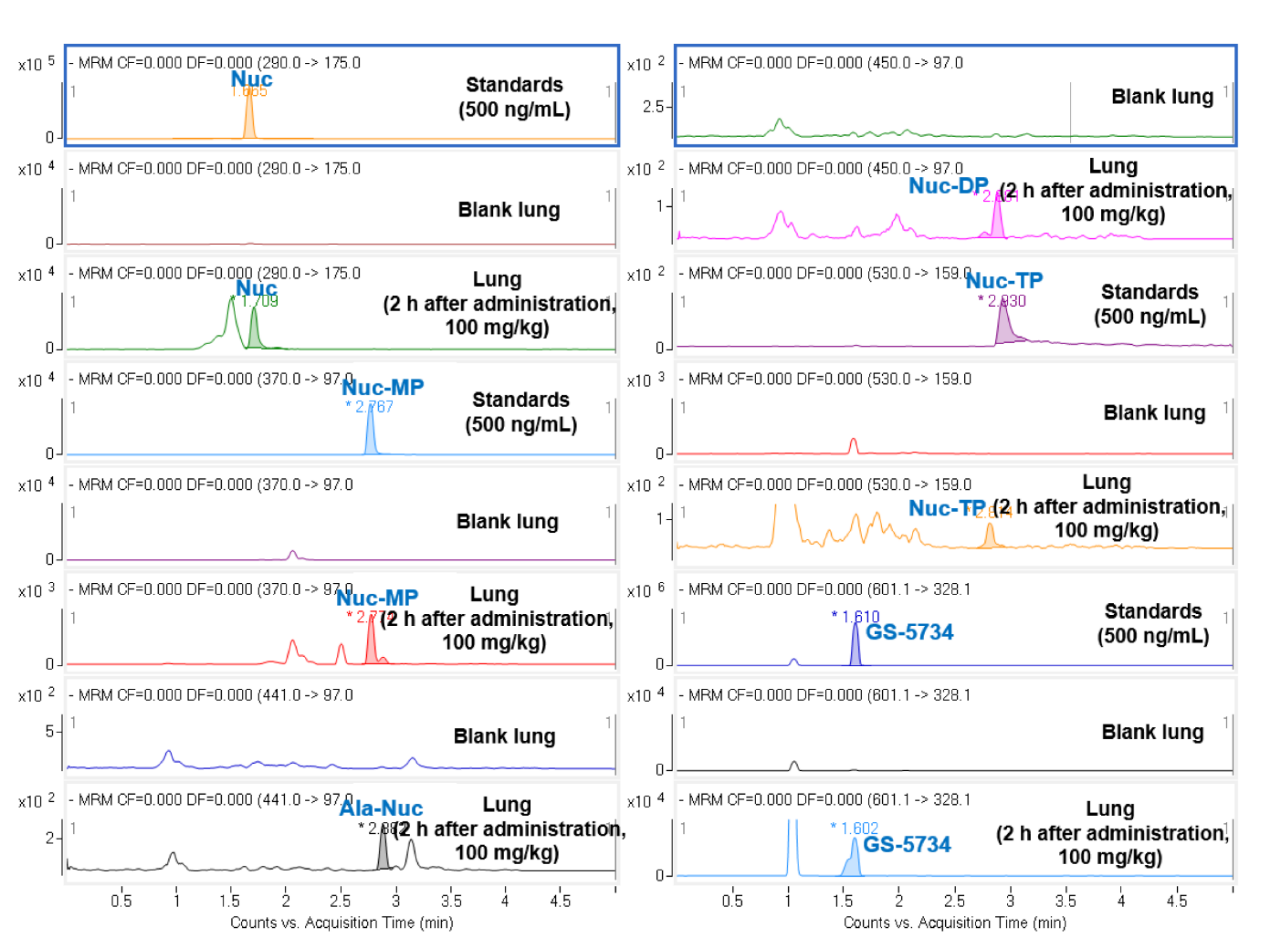
 **Figure. S3.** **UHPLC-MS/MS chromatograms of remdesivir and its metabolites in rat lung and standard solution.**


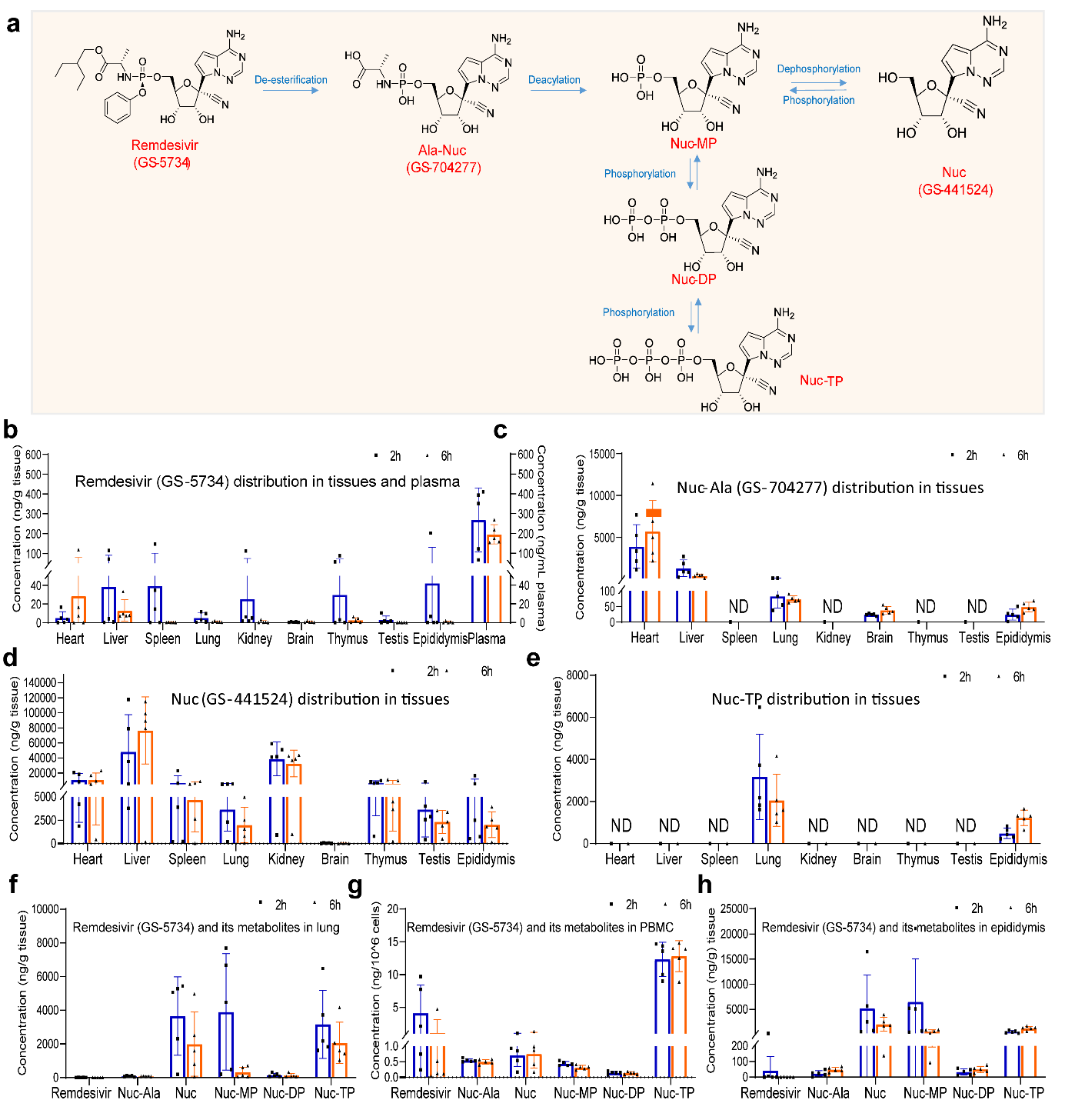


**Figure. S4. Tissue distribution and metabolism of remdesivir after ip injection in rats.** 10 male Sprague-Dawley rats were administered with GS-5734 at a single dose of 100 mg/kg, i.p. At the time point of 2 h and 6 h after drug administration, five rats were dissected. The blood samples as well as heart, liver, spleen, lung, kidney, brain, thymus, testis, and epididymis were collected for UHPLC-MS/MS analysis performed on a UHPLC system (1290 series, Agilent Technologies, US) coupled to a triple quadrupole mass spectrometer (Agilent 6470 QQQ). **a**. Metabolic pathway and key metabolites of remdesivir. **b**. Remdesivir (GS-5734) distribution in tissues and plasma (100 mg/kg). **c**. Ala-Nuc (GS-704277) distribution in tissues (100 mg/kg). **d**. Nuc (GS-441524) distribution in tissues (100 mg/kg). **e**. Nuc-TP distribution in tissues (100 mg/kg). **f**. Remdesivir (GS-5734) and its metabolites in lung (100 mg/kg). **g**. Remdesivir (GS-5734) and its metabolites in PBMCs (100 mg/kg). **h**. Remdesivir (GS-5734) and its metabolites in epididymis (100 mg/kg). ND: not detectable.


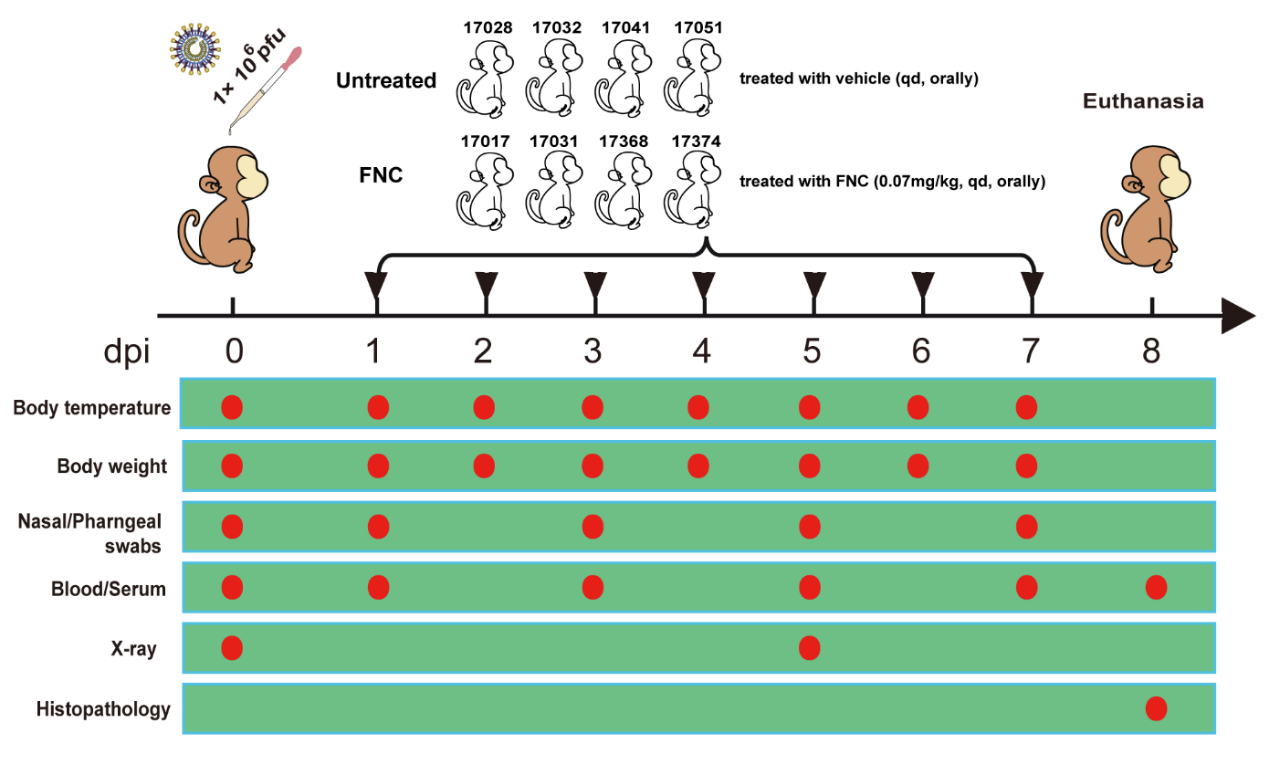
 **Figure. S5.** **Schematic diagram of the study design.** Eight 3-4 years old rhesus macaques (RM) monkeys (four males and four females) were randomly enrolled in this study. All animals were inoculated with SARS-CoV-2, 1×10^6^ pfu, following treated with vehicle or FNC, as stated in Materials and Methods. Clinical signs, viral load, hematology, blood biochemical indications, chest radiograph and histological evaluation were conducted at the indicated time points.


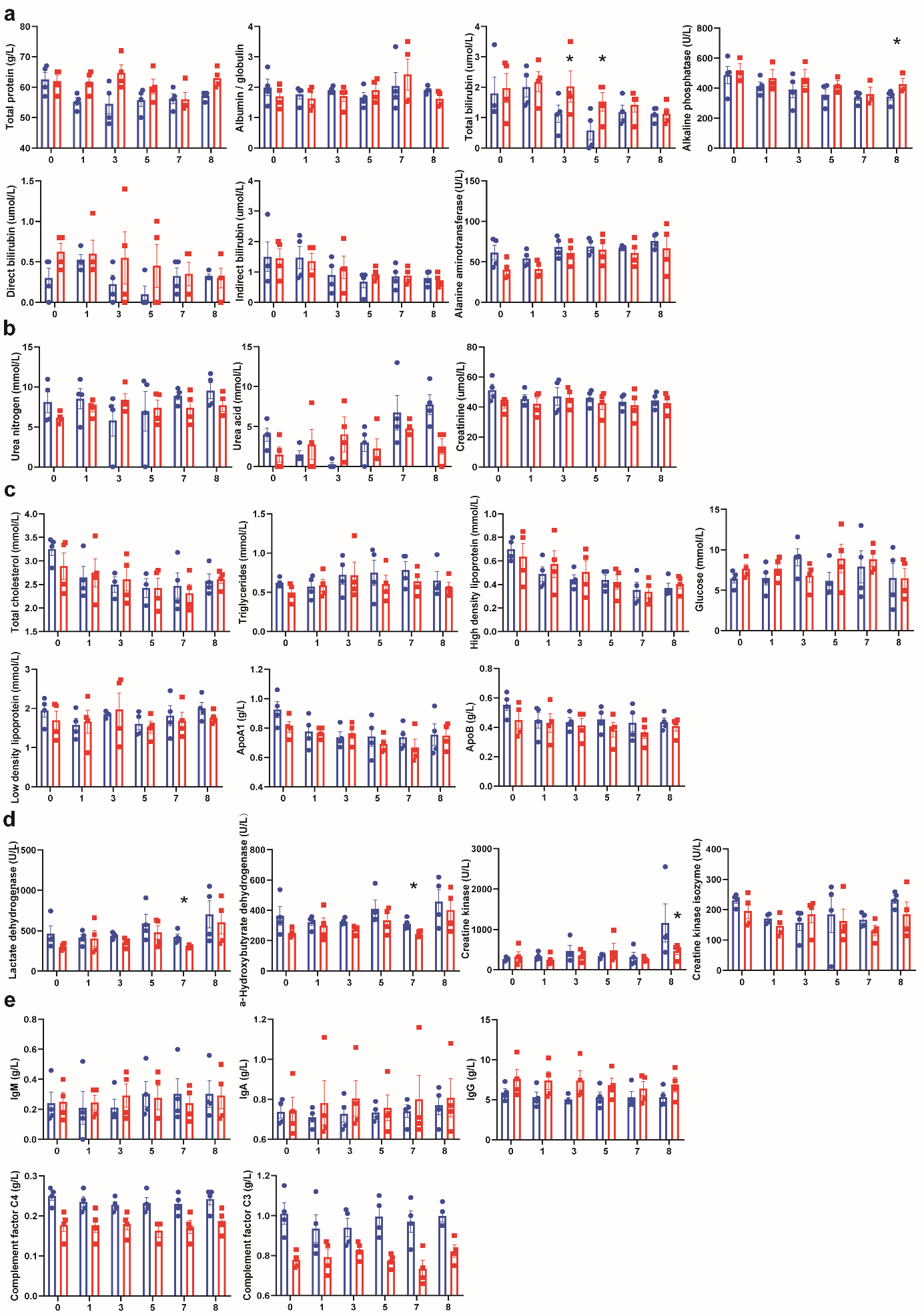
 **Figure. S6.** **Effect of FNC on peripheral blood indications after SARS-CoV-2 infection**. Anti-coagulant blood from inguinal veins of anaesthetized monkeys was collected for plasma preparation. Biochemical indications in plasma were measured on the automatic biochemistry analyzer. **a**. Liver function related indications. **b**. Kidney function related indications. **c**. Lipid and glucose related factors. **d**. Heart and muscle related indications. E. Immunity related factors. Data are presented as mean ± SEM (n = 4). *p< 0.05, monkey in FNC group (red bar) vs. monkey in untreated group (blue bar) by Mann-Whitney U test.


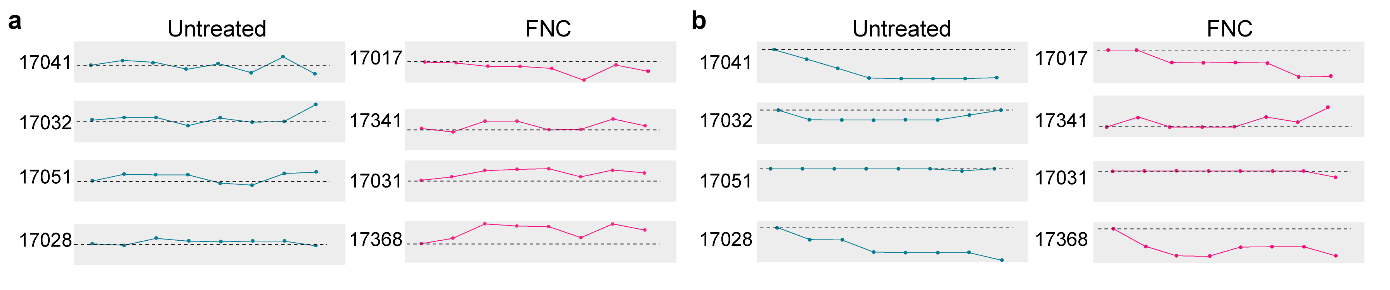
 **Figure. S7.** **Changes of body temperature and body weight.** Body temperature and body weight of the monkeys were measured every day from 0 dpi. The changes are normalized to 0 dpi (baseline). **a**. Temperature change to 0 dpi. **b**. Body weight change to 0 dpi.


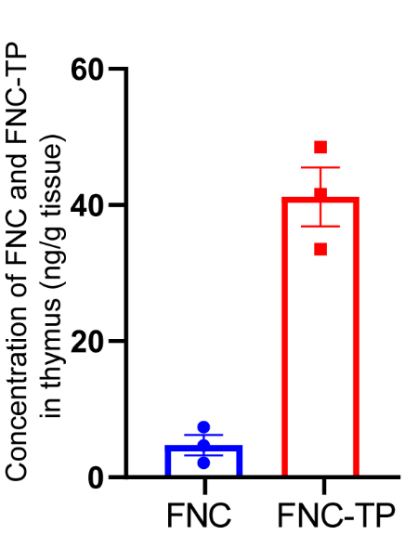


**Figure. S8.** **FNC-TP accumulated in the thymus tissues of RM monkey.** By the end of the experiment, 6 hrs after drug administration, the RM monkeys were practiced euthanasia, and the thymus samples were collected for UHPLC-MS/MS analysis, operated on a HPLC system (1290 series, Agilent Technologies, US) coupled to a triple quadrupole mass spectrometer (Agilent 6470 QQQ).


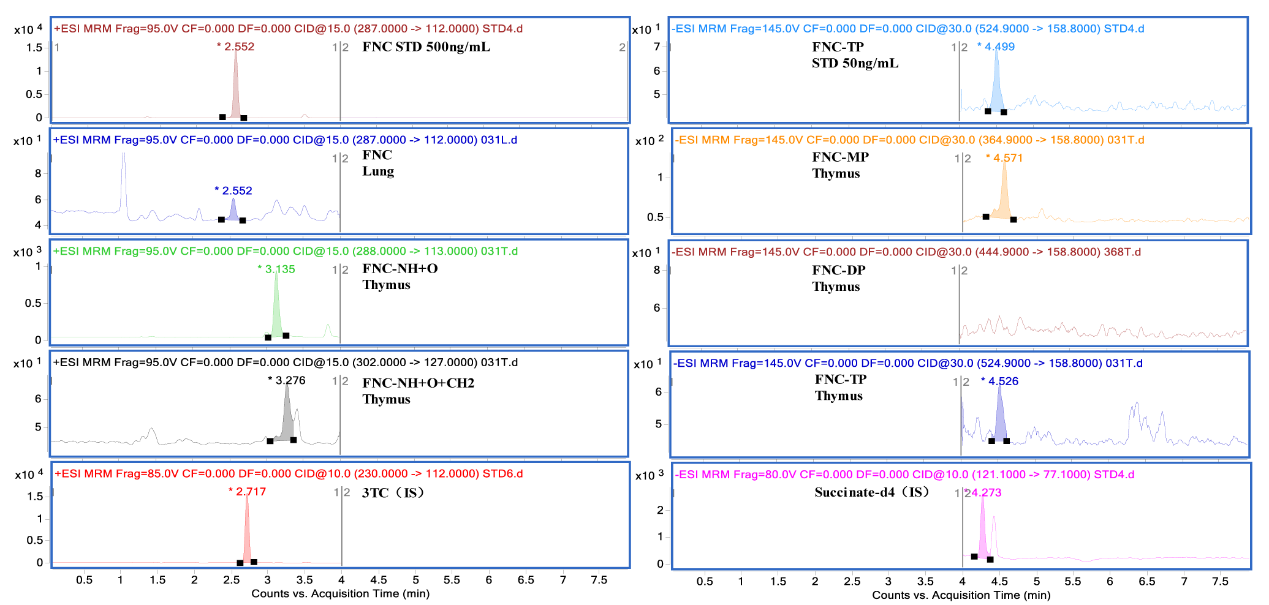
 **Figure. S9.** **UHPLC-MS/MS chromatograms of FNC and its metabolites in thymus of RM monkeys and standard solution.** 3TC (lamivudine) was used as an internal control.


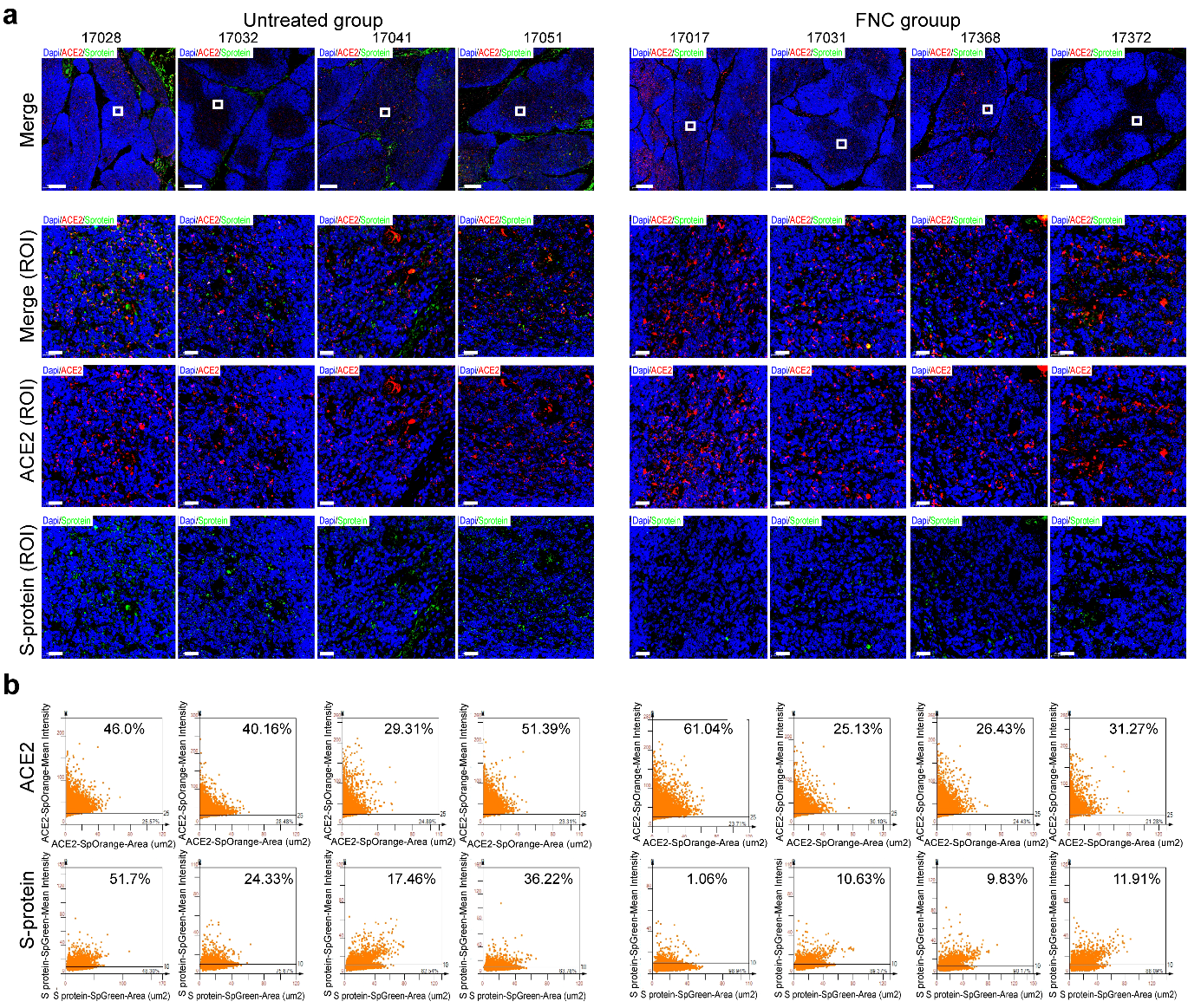
 **Figure. S10. FNC selectively protected thymus from SARS-CoV-2 infection. a**. The representative images of multi-color immune fluorescent staining for ACE2 (red) and S protein (green) in the thymus from RM monkeys inoculated with SARS-CoV-2 with (right group) or without FNC treatment (left group). The regions of interest (ROI) are boxed in white, and their magnified photos are shown below. Scale bars, 200 µm (up) and 20 µm (down). **b**. The % of ACE2+ cells (up) or S protein+ cells (down) in thymus of the SARS-CoV-2 inoculated RM monkeys were analyzed using Tissue FAXS platform and Tissue Quest software (Tissue Gnostics). A, second line: ROI merge.


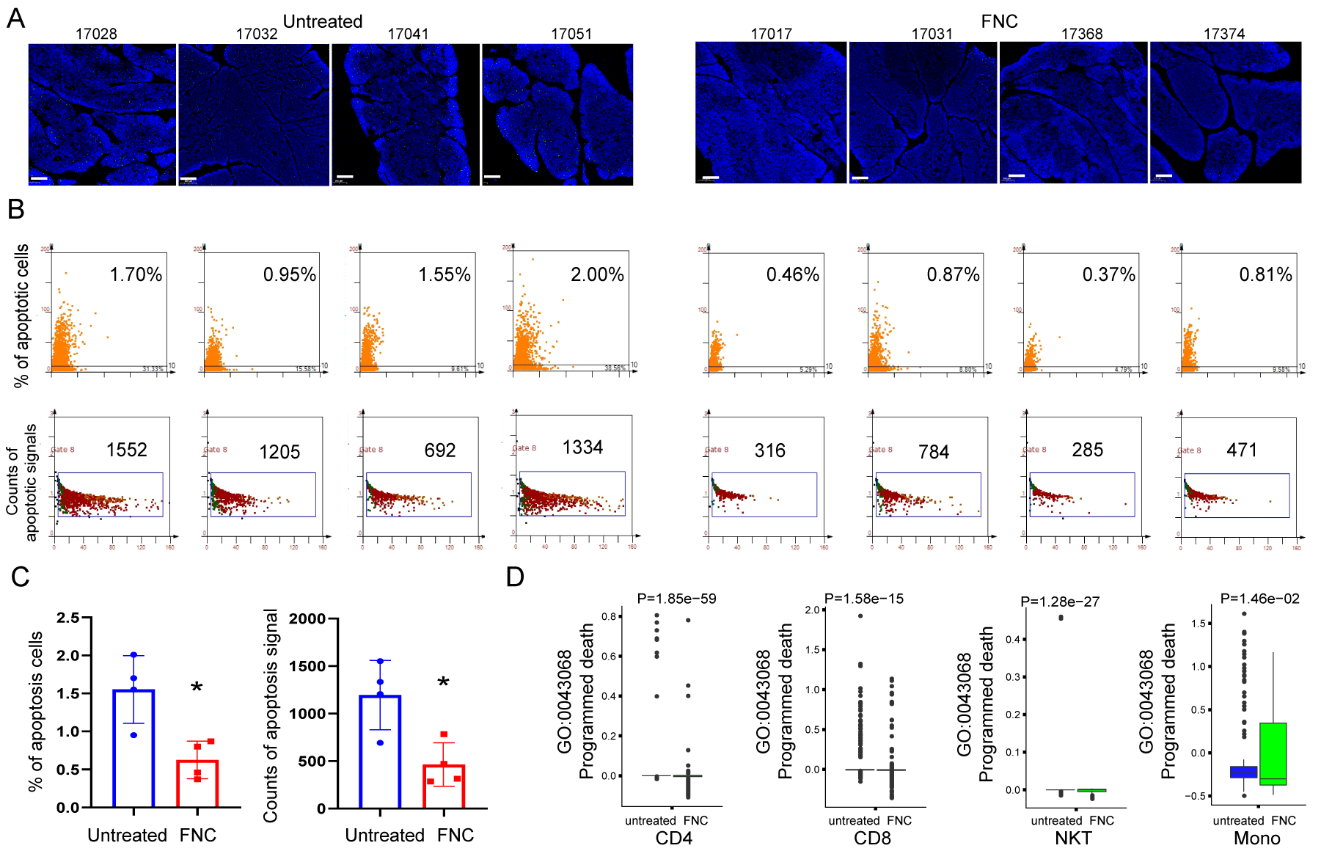


**Figure. S11. FNC might reduce apoptosis in thymus after SARS-CoV-2 infection.** Relative proportion and absolute numbers of apoptotic cells in thymus of SARS-CoV-2 inoculated RM monkeys with or without FNC treatment. **a**. Fluorescent images of tunnel staining in thymus of the study monkey. **b**. Apoptotic cells were evaluated using Tissue FAXS platform and Tissue Quest software (Tissue Gnostics). **c**. Statistics (n=4, for both groups) was done for the relative proportion of apoptosis cells and the numbers of apoptotic signals in thymus, based on the results in B (* p< 0.05). Scale bars, 200 µm. **d**. Histogram of expression levels of GO biological process terms apoptosis and programmed death, in subset cells from infected RM monkeys treated and untreated with FNC. Horizontal lines represent median values, and statistics are on the top of each figure. Blue box: untreated (code# 17041); green box: FNC treated (code #17368).

**
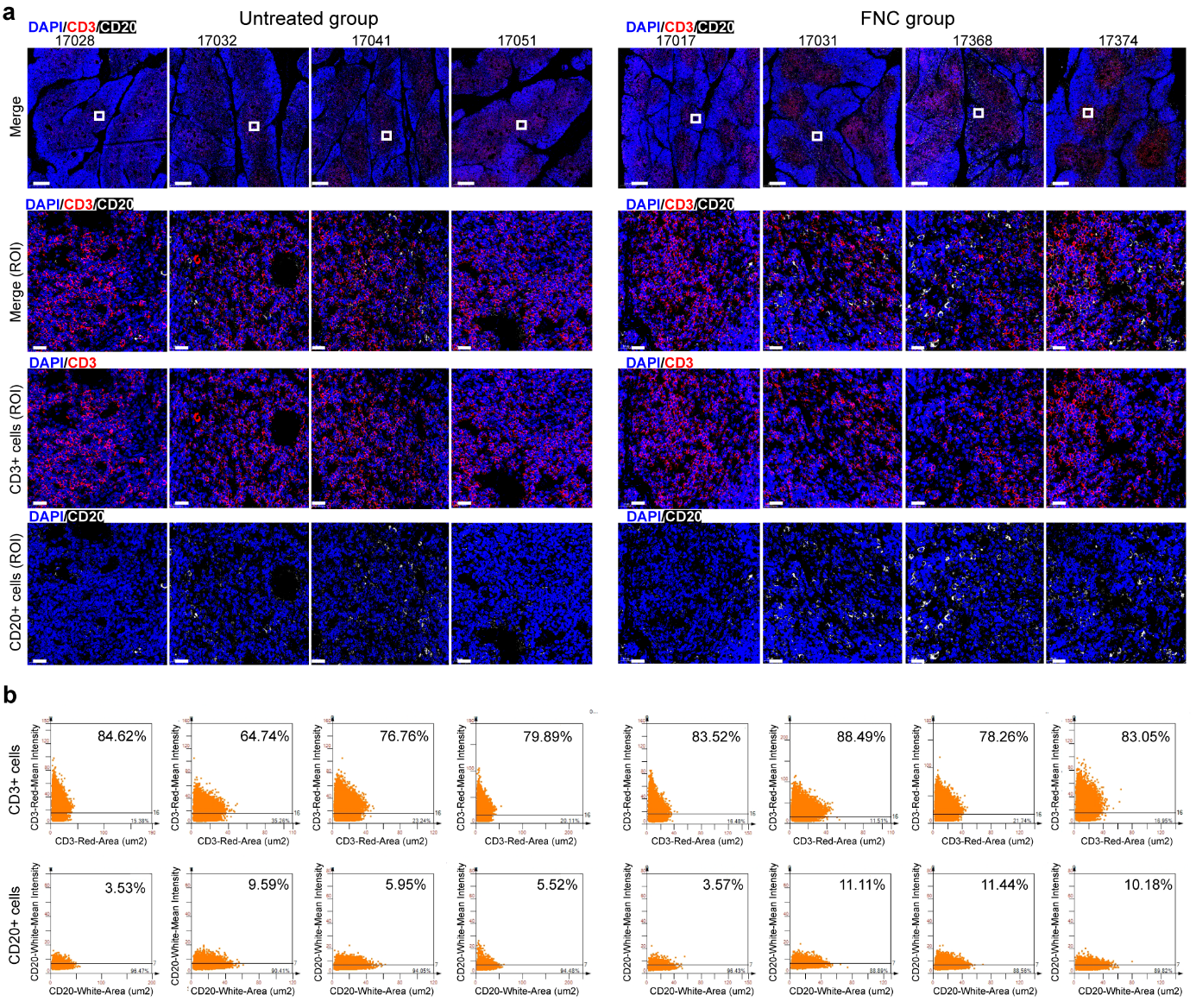
 Figure. S12. FNC improves immune cell profile in thymus of the viral infected RM monkeys. a**. Representative images of multi-color immunofluorescent staining was for CD3 (red) and CD20 (white) protein in the thymus of the RM monkeys inoculated with SARS-CoV-2, treated or untreated with FNC (Day 8 samples). The regions of interest (ROI) are boxed in white (up), and their magnified photos are shown below (merged ROI). Scale bars, 200 µm (up) and 20 µm (down). **b**. Relative proportion of CD3+ and CD20+ cells in the thymus of the study RM monkeys were analyzed using Tissue FAXS platform and Tissue Quest software (Tissue Gnostics).

**
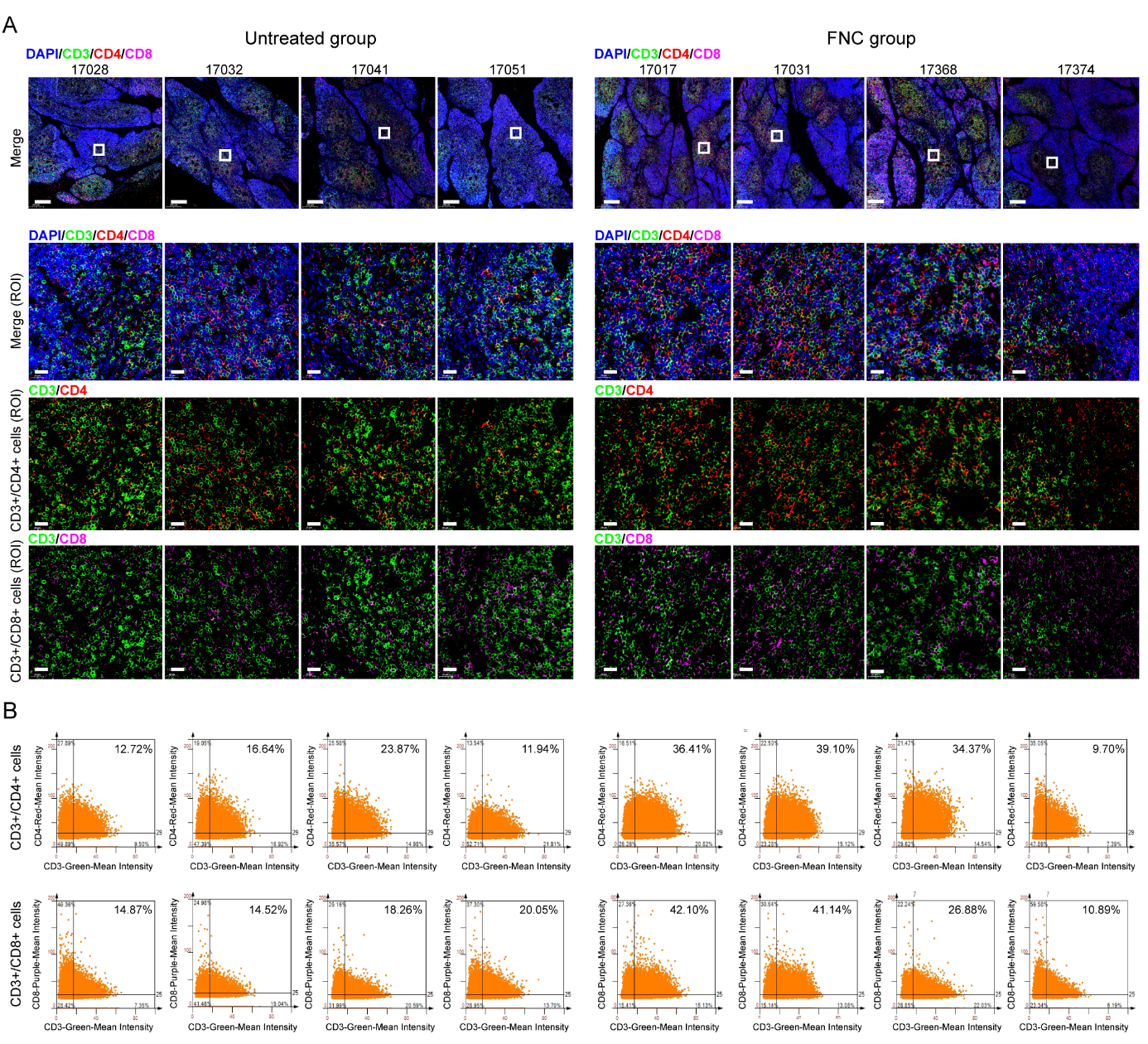
 Figure. S13. Fig FNC improves T-cell profile in thymus of the study RM monkeys. a**. Representative images of multi-color immune-fluorescent staining was done for CD3 (green), CD4 (red) and CD8 (purple) protein in the thymus of the RM monkeys inoculated with SARS-CoV-2, treated or untreated with FNC (monkey code is shown on the top of the figure). The regions of interest (ROI) are boxed in white (up), and their magnified photos are shown below. Scale bars, 200 µm (up) and 20 µm (down). **b**. Relative proportion of CD3+/CD4+ cells and CD3+/CD8+ cells in thymus of the SARS-CoV-2 inoculated RM monkeys with or without FNC treatment was analyzed using Tissue FAXS platform and Tissue Quest software (Tissue Gnostics).


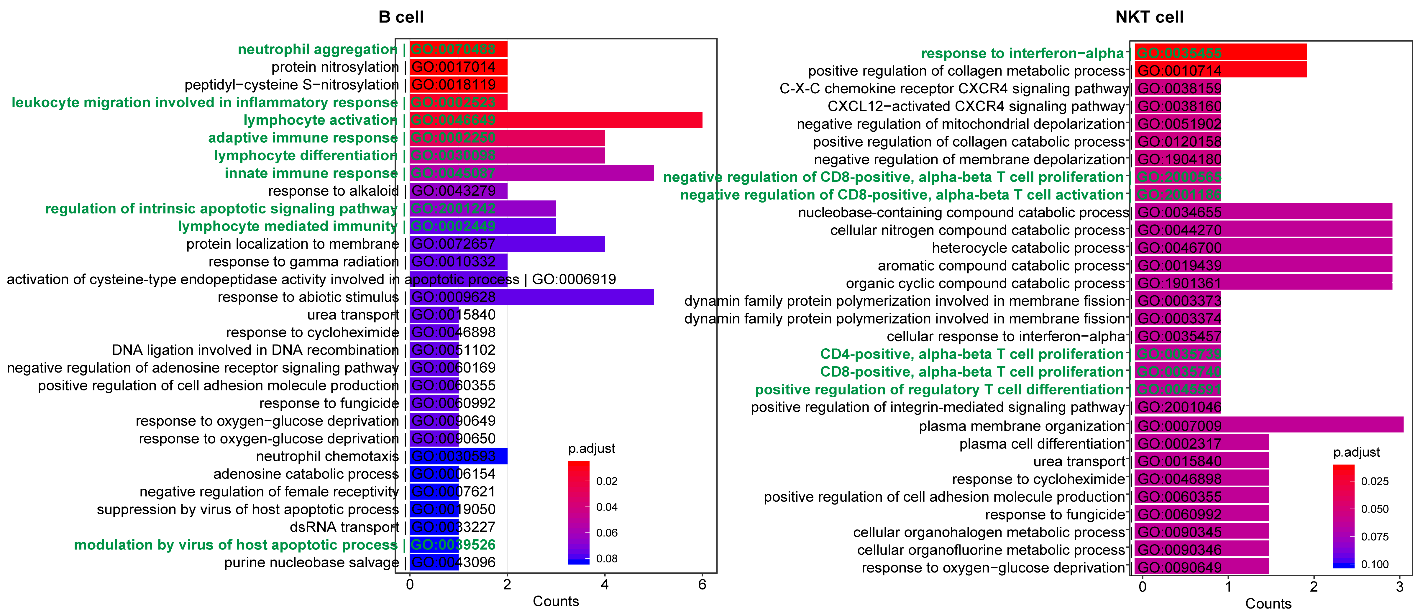
 **Figure. S14.** **Top 30 enriched GO terms in the subset of B and NKT.** GO terms are labeled with name and ID, and the red-to-blue color represents the level of P value. Interesting terms are labeled in green.


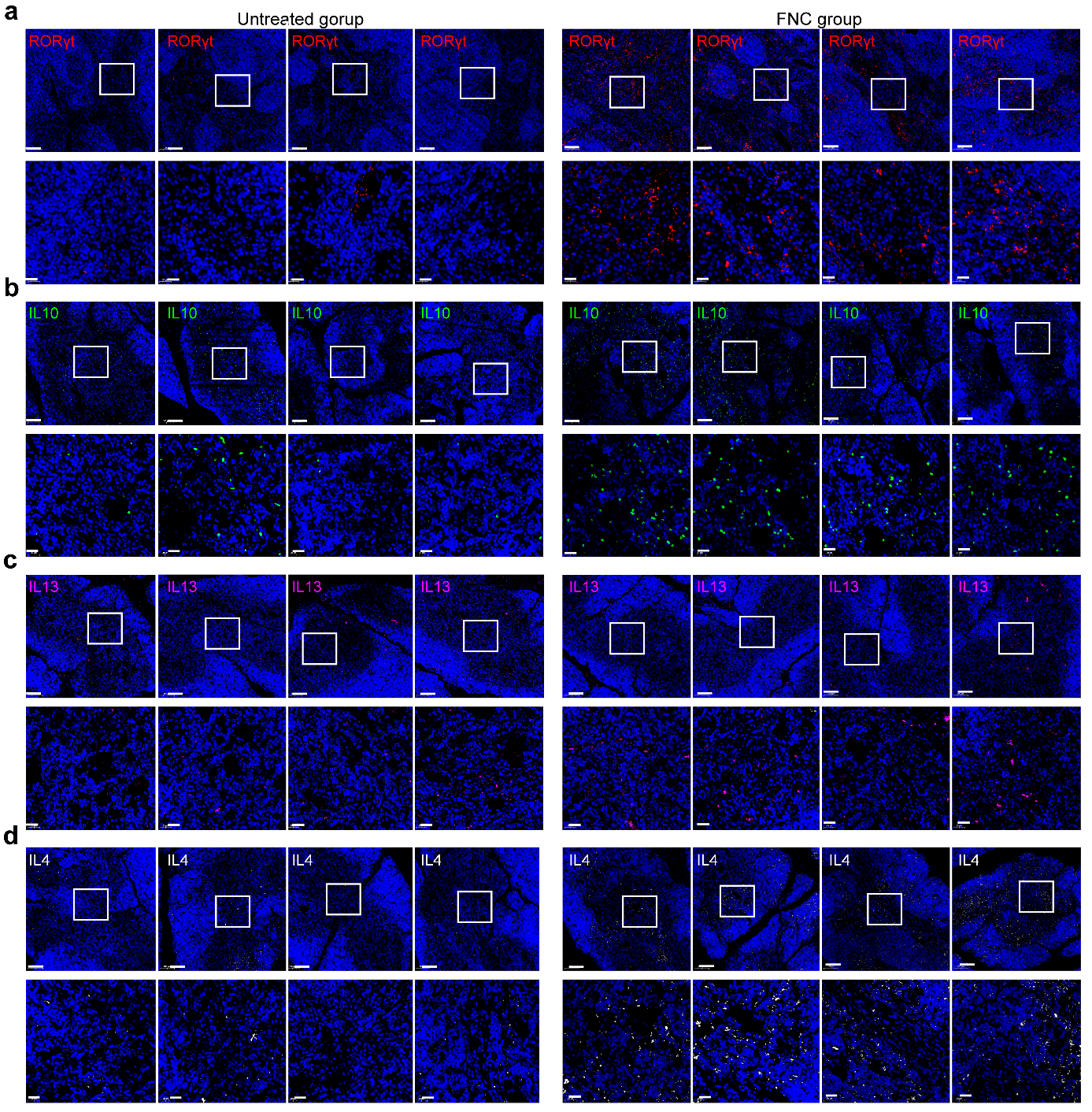
 **Figure. S15. Immuno-fluorescent staining for immune markers in thymus of RM monkeys inoculated with SARS-CoV-2, treated or untreated with FNC.** **a**. RORγt (red); **b**. IL10 (green); **c**. IL13 (purple); **d**. IL4 (white). The regions of interest (ROI) are boxed in white (up), and their magnified photos are shown below. Scale bars, 200 µm (up) and 20 µm (down).

**Table S1. Distribution of FNC and its metabolites after oral administration in rats**

Table S1a. FNC distribution in tissues and plasma

| Concentration (ng/g tissue) | 1 h | | 2 h | | 6 h | | 12 h | | 24 h | |
| --- | --- | --- | --- | --- | --- | --- | --- | --- | --- | --- |
|  | Average | STDEV | Average | STDEV | Average | STDEV | Average | STDEV | Average | STDEV |
| Heart | 237.93 | 129.43 | 265.04 | 93.29 | 132.72 | 32.52 | 38.81 | 15.49 | 1.76 | 0.75 |
| Liver | 276.94 | 188.54 | 229.10 | 50.36 | 151.78 | 132.96 | 103.99 | 47.73 | 14.68 | 7.14 |
| Spleen | 928.18 | 223.42 | 870.92 | 391.97 | 939.92 | 561.94 | 591.66 | 233.85 | 351.57 | 418.91 |
| Lung | 362.95 | 202.74 | 268.95 | 63.64 | 211.76 | 106.21 | 128.41 | 68.84 | 42.00 | 15.11 |
| Kidney | 515.36 | 307.56 | 797.61 | 320.28 | 269.07 | 145.75 | 76.54 | 14.45 | 78.09 | 67.37 |
| Brain | 214.87 | 153.90 | 206.81 | 63.90 | 149.85 | 37.25 | 54.96 | 4.70 | 14.74 | 2.92 |
| Thymus | 1191.54 | 264.87 | 1481.50 | 736.86 | 2585.30 | 1483.06 | 2019.23 | 367.77 | 499.72 | 184.28 |
| Testis | 711.78 | 399.85 | 343.60 | 203.91 | 189.18 | 138.20 | 67.87 | 18.26 | 15.14 | 2.64 |
| Epididymis | 135.52 | 32.35 | 506.81 | 312.64 | 84.34 | 56.47 | 40.96 | 18.51 | 12.34 | 4.22 |
| Plasma (ng/mL) | 681.83 | 49.46 | 673.23 | 26.78 | 211.63 | 65.41 | 59.36 | 7.68 | 26.63 | 10.03 |

Rat, n=5

STDEV: standard deviation

Table S1b. FNC-TP levels in blood

| Concentration | 1 h | | 2 h | | 6 h | | 12 h | | 24 h | |
| --- | --- | --- | --- | --- | --- | --- | --- | --- | --- | --- |
|  | Average | STDEV | Average | STDEV | Average | STDEV | Average | STDEV | Average | STDEV |
| Plasma (ng/mL) | ND | ND | ND | ND | ND | ND | ND | ND | ND | ND |
| PBMCs (ng/10^6 cells) | 0.29 | 0.08 | 0.32 | 0.09 | 0.19 | 0.03 | 0.93 | 0.91 | 0.52 | 0.21 |

Rat, n=5

ND: not detectable.

PBMCs: Peripheral blood mononuclear cells

Table S1c. FNC-TP levels in tissues

| Concentration (ng/g tissue) | 1 h | | 2 h | | 6 h | | 12 h | | 24 h | |
| --- | --- | --- | --- | --- | --- | --- | --- | --- | --- | --- |
|  | Average | STDEV | Average | STDEV | Average | STDEV | Average | STDEV | Average | STDEV |
| Heart | ND | ND | ND | ND | ND | ND | ND | ND | ND | ND |
| Liver | ND | ND | ND | ND | ND | ND | ND | ND | ND | ND |
| Spleen | ND | ND | ND | ND | ND | ND | ND | ND | ND | ND |
| Lung | ND | ND | ND | ND | ND | ND | ND | ND | ND | ND |
| Kidney | ND | ND | ND | ND | ND | ND | ND | ND | ND | ND |
| Brain | ND | ND | ND | ND | ND | ND | ND | ND | ND | ND |
| Thymus | 199.10 | 51.02 | 191.97 | 57.43 | 133.07 | 57.05 | 115.85 | 27.70 | 141.44 | 65.83 |
| Testis | ND | ND | ND | ND | ND | ND | ND | ND | ND | ND |
| Epididymis | ND | ND | ND | ND | ND | ND | ND | ND | ND | ND |
| Plasma (ng/mL) | ND | ND | ND | ND | ND | ND | ND | ND | ND | ND |

Rat, n=5

ND: not detectable.

Table S1d. FNC & its phosphate metabolites (FNC-MP, FNC-DP and FNC-TP) in thymus

| Concentration (ng/g tissue) | 1 hr | | 2 hr | | 6 hr | | 12 hr | | 24 hr | |
| --- | --- | --- | --- | --- | --- | --- | --- | --- | --- | --- |
|  | Average | STDEV | Average | STDEV | Average | STDEV | Average | STDEV | Average | STDEV |
| FNC | 1191.54 | 264.87 | 1481.50 | 736.86 | 2585.30 | 1483.06 | 2019.23 | 367.77 | 499.72 | 184.28 |
| FNC-MP | 742.26 | 187.99 | 727.11 | 219.92 | 476.59 | 223.52 | 513.53 | 86.15 | 510.07 | 280.96 |
| FNC-DP | 477.83 | 139.91 | 387.76 | 141.65 | 294.44 | 87.32 | 216.92 | 25.82 | 316.40 | 147.62 |
| FNC-TP | 199.10 | 51.02 | 191.97 | 57.43 | 133.07 | 57.05 | 115.85 | 27.70 | 141.44 | 65.83 |

Rat, n=5

Table S1e. FNC & its phosphate metabolites in PBMCs

| Concentration (ng/10^6 cells) | 1 h | | 2 h | | 6 h | | 12 h | | 24 h | |
| --- | --- | --- | --- | --- | --- | --- | --- | --- | --- | --- |
|  | Average | STDEV | Average | STDEV | Average | STDEV | Average | STDEV | Average | STDEV |
| FNC | 1.56 | 0.39 | 1.79 | 0.84 | 0.28 | 0.08 | 0.10 | 0.03 | 0.49 | 0.26 |
| FNC-MP | 0.93 | 0.11 | 1.07 | 0.12 | 0.80 | 0.21 | 1.56 | 0.87 | 1.44 | 0.61 |
| FNC-DP | 0.98 | 0.39 | 0.99 | 0.44 | 0.50 | 0.10 | 2.99 | 0.62 | 2.46 | 1.78 |
| FNC-TP | 0.29 | 0.08 | 0.32 | 0.09 | 0.19 | 0.03 | 0.93 | 0.91 | 0.52 | 0.21 |

Rat, n=5

**Table S2. Distribution of Remdesivir and its metabolites after ip injection in rats**

Supplementary Tab 2a Remdesivir (GS-5734) distribution in tissues and plasma

| Concentration (ng/g tissue) | | Heart | Liver | Spleen | Lung | Kidney | Brain | Thymus | Testis | Epididymis | Plasma (mg/mL) |
| --- | --- | --- | --- | --- | --- | --- | --- | --- | --- | --- | --- |
| 2 h | Average | 5.12 | 38.43 | 39.36 | 5.14 | 25.44 | 0.61 | 29.87 | 2.88 | 42.06 | 269.23 |
|  | STDEV | 6.73 | 52.46 | 61.84 | 5.23 | 48.94 | 0.30 | 40.85 | 4.29 | 89.89 | 161.43 |
| 6 h | Average | 28.58 | 13.05 | 0.32 | 0.87 | 0.84 | 0.78 | 2.74 | 0.11 | 0.60 | 196.37 |
|  | STDEV | 50.84 | 11.44 | 0.25 | 0.68 | 1.29 | 0.68 | 2.64 | 0.12 | 0.84 | 48.36 |

Rat, n=5

Table S2b. Ala-Nuc (GS-704277) distribution in tissues

| Concentration (ng/g tissue) | | Heart | Liver | Spleen | Lung | Kidney | Brain | Thymus | Testis | Epididymis |
| --- | --- | --- | --- | --- | --- | --- | --- | --- | --- | --- |
| 2 h | Average | 3908.49 | 1304.94 | ND | 82.87 | ND | 24.16 | ND | ND | 24.05 |
|  | STDEV | 2612.43 | 989.83 | ND | 37.58 | ND | 3.62 | ND | ND | 17.69 |
| 6 h | Average | 5730.68 | 416.97 | ND | 73.42 | ND | 38.27 | ND | ND | 48.37 |
|  | STDEV | 3669.12 | 186.73 | ND | 10.73 | ND | 11.95 | ND | ND | 15.58 |

Rat, n=5; ND: not detectable

Table S2c. Nuc (GS-441524) distribution in tissues

| Concentration (ng/g tissue) | | Heart | Liver | Spleen | Lung | Kidney | Brain | Thymus | Testis | Epididymis |
| --- | --- | --- | --- | --- | --- | --- | --- | --- | --- | --- |
| 2 h | Average | 10667.09 | 48271.59 | 6772.452 | 3658.072 | 38705.51 | 45.798 | 6366.984 | 3661.204 | 5185.526 |
|  | STDEV | 8394.769 | 49355.36 | 9490.462 | 2327.688 | 22364.52 | 27.89274 | 3390.459 | 2949.059 | 6635.958 |
| 6 h | Average | 10987.00 | 76606.35 | 4672.95 | 1984.53 | 32594.46 | 38.44 | 5851.81 | 2335.77 | 2022.44 |
|  | STDEV | 9011.05 | 44693.47 | 3406.32 | 1904.77 | 17865.85 | 26.72 | 4494.41 | 1230.34 | 1378.78 |

Rat, n=5

Table S2d. Nuc-TP distribution in tissues

| Concentration (ng/g tissue) | | Heart | Liver | Spleen | Lung | Kidney | Brain | Thymus | Testis | Epididymis |
| --- | --- | --- | --- | --- | --- | --- | --- | --- | --- | --- |
| 2 h | Average | ND | ND | ND | 3168.31 | ND | ND | ND | ND | 486.87 |
|  | STDEV | ND | ND | ND | 2029.16 | ND | ND | ND | ND | 245.05 |
| 6 h | Average | ND | ND | ND | 2057.66 | ND | ND | ND | ND | 1225.27 |
|  | STDEV | ND | ND | ND | 1232.30 | ND | ND | ND | ND | 367.06 |

Rat, n=5

ND: not detectable

Table S2e. Remdesivir (GS-5734) and its metabolites in lung

| Concentration (ng/g tissue) | | Remdesivir | Ala-Nuc | Nuc | Nuc-MP | Nuc-DP | Nuc-TP |
| --- | --- | --- | --- | --- | --- | --- | --- |
| 2 h | Average | 5.14 | 82.87 | 3658.07 | 3904.37 | 146.16 | 3168.31 |
|  | STDEV | 5.23 | 37.58 | 2327.69 | 3456.76 | 115.09 | 2029.16 |
| 6 h | Average | 0.87 | 73.42 | 1984.53 | 309.88 | 118.94 | 2057.66 |
|  | STDEV | 0.68 | 10.73 | 1904.77 | 321.70 | 112.56 | 1232.30 |

Rat, n=5

Table S2f. Remdesivir (GS-5734) and its metabolites in PBMCs

| Concentration (ng/10^6 cells) | | Remdesivir | Ala-Nuc | Nuc | Nuc-MP | Nuc-DP | Nuc-TP |
| --- | --- | --- | --- | --- | --- | --- | --- |
| 2 h | Average | 4.11 | 0.55 | 0.71 | 0.44 | 0.15 | 12.32 |
|  | STDEV | 4.32 | 0.05 | 0.35 | 0.07 | 0.04 | 2.63 |
| 6 h | Average | 1.10 | 0.50 | 0.75 | 0.32 | 0.14 | 12.82 |
|  | STDEV | 2.03 | 0.08 | 0.45 | 0.07 | 0.04 | 2.35 |

Rat, n=5

PBMCs: Peripheral blood mononuclear cells

Table S2g. Remdesivir (GS-5734) and its metabolites in epididymis

| Concentration (ng/g tissue) | | Remdesivir | Ala-Nuc | Nuc | Nuc-MP | Nuc-DP | Nuc-TP |
| --- | --- | --- | --- | --- | --- | --- | --- |
| 2 h | Average | 42.06 | 24.05 | 5185.53 | 6493.99 | 33.09 | 486.87 |
|  | STDEV | 89.89 | 17.69 | 6635.96 | 8482.61 | 20.30 | 245.05 |
| 6 h | Average | 0.60 | 48.37 | 2022.44 | 555.72 | 49.65 | 1225.27 |
|  | STDEV | 0.84 | 15.58 | 1378.78 | 362.55 | 20.05 | 367.06 |

Rat, n=5

**Table S3. Clinical information for individual COVID-19 patient of the cohort**

| No | Hospital | Gender | Age | Severity | Drug intervention  history | Time from virus positive  to FNC treatment (Days) | Time from FNC treatment to  SARS-CoV-2 negative conversion (Days) | Dosage | Duration of FNC treatment (Days) | Combination  treatment | Time from FNC treatment to  discharge from the hospital (Days) | Side effect |
| --- | --- | --- | --- | --- | --- | --- | --- | --- | --- | --- | --- | --- |
| 1 | HPPH | Female | 56 | Severe | Anti-virus, TCM | 13 | 3 | 10mg, QD×1  5mg, QD | 4 | INFα+TCM | 5 | Dizziness, Nausea,  Vomit (first day) |
| 2 | HPPH | Female | 71 | Severe | Antivirus, TCM | 37 | 8 | 10mg, QD×1  5mg, QD | 9 | INFα+TCM | 10 | Dizziness, Insomnia,  Vomit (first day) |
| 3 | HPPH | Female | 31 | Severe | Anti-virus, Anti-bacteria | 4 | 2 | 10mg, QD×1  5mg, QD | 2 |  | 2 |  |
| 4 | HPPH | Female | 26 | Severe | Anti-virus,  Anti-bacteria, TCM | 16 | 1 | 10mg, QD×1  5mg, QD | 2 |  | 2 |  |
| 5 | HPPH | Male | 23 | Moderate |  | 3 | 4 | 10mg, QD×1  5mg, QD | 9 | TCM | 8 |  |
| 6 | HPPH | Male | 28 | Moderate |  | 3 | 6 | 10mg, QD×1  5mg, QD | 6 |  | 9 |  |
| 7 | HPPH | Male | 47 | Moderate |  | 3 | 9 | 10mg, QD×1  5mg, QD | 10 | TCM | 13 |  |
| 8 | HPPH | Male | 28 | Moderate |  | 3 | 4 | 10mg, QD×1  5mg, QD | 10 | TCM | 11 |  |
| 9 | FAH | Male | 45 | Moderate | Anti-virus | 13 | 3 | 5mg, QD | 6 | INFα+ Abdol+ Lopinavir/ritonavir | 7 |  |
| 10 | FAH | Female | 32 | Moderate | Anti-virus, TCM | 8 | 3 | 5mg, QD | 5 |  | 5 |  |
| 11 | FAH | Female | 20 | Moderate | Anti-virus | 3 | 4 | 5mg, QD | 3 | Lopinavir/ritonavir | 10 |  |
| 12 | FAH | Female | 47 | Moderate | Anti-virus, TCM | 10 | 8 | 5mg, QD | 10 | INFα+TCM | 12 |  |
| 13 | FAH | Female | 52 | Moderate | Anti-virus,  Anti-bacteria, TCM | 11 | 3 | 5mg, QD | 6 | Chloroquine+  Thymosin | 6 |  |
| 14 | FAH | Female | 77 | Moderate | Anti-virus, Anti-bacteria, TCM | 16 | 6 | 5mg, QD | 6 | Chloroquine+  Thymosin+  TCM | 8 |  |
| 15 | FAH | Female | 26 | Moderate | Anti-virus, TCM | 21 | 1 | 5mg, QD | 2 | Abdol+ Lopinavir/ritonavir | 2 |  |
| 16 | FAH | Female | 40 | Moderate | Anti-virus | 31 | 1 | 5mg, QD | 7 | TCM | 9 |  |
| 17 | FAH | Female | 33 | Moderate | Anti-virus | 30 | 6 | 5mg, QD | 7 | INFα+TCM+  Abdol | 8 |  |
| 18 | ZHWU | Female | 58 | Moderate | Anti-virus,  TCM | 32 | 4 | 5mg, QD | 12 | TCM | 13 |  |
| 19 | ZHWU | Male | 68 | Moderate | Anti-virus,  Anti-bacteria, TCM | 30 | 3 | 5mg, QD | 10 | TCM | 16 |  |
| 20 | ZHWU | Female | 69 | Moderate | Anti-virus, Anti-bacteria, | 47 | 1 | 5mg, QD | 3 |  | 3 |  |
| 21 | ZHWU | Female | 70 | Moderate | Anti-virus, TCM | 24 | 3 | 5mg, QD | 6 | TCM+ Abidol | 11 | Dizziness (fist day) |
| 22 | ZHWU | Female | 81 | Moderate | Anti-virus, Anti-bacteria, TCM | 28 | 3 | 5mg, QD | 8 |  | 25 |  |
| 23 | ZHWU | Female | 61 | Moderate | Anti-virus, Anti-bacteria, TCM | 25 | 2 | 5mg, QD | 12 |  | 19 |  |
| 24 | ZHWU | Male | 69 | Severe | Anti-virus, Anti-bacteria, TCM | 36 | 1 | 5mg, QD | 8 |  | 9 |  |
| 25 | ZHWU | Female | 79 | Moderate | Unknown | 47 | 3 | 5mg, QD | 7 |  | 6 |  |
| 26 | ZHWU | Female | 58 | Moderate | Unknown | 26 | 2 | 5mg, QD | 7 |  | 6 |  |
| 27 | ZHWU | Female | 29 | Moderate | Anti-virus,  TCM | 14 | 1 | 5mg, QD | 5 |  | 6 | Nausea, Vomit (first day) |
| 28 | ZHWU | Female | 51 | Moderate | Anti-virus, Anti-bacteria, TCM | 59 | 2 | 5mg, QD | 6 |  | 12 | Low counts of Platelet and Fibrinogen (first  day) |
| 29 | ZHWU | Male | 76 | Moderate | Anti-virus, Anti-bacteria, TCM | 45 | 1 | 5mg, QD | 8 |  | 10 |  |
| 30 | ZHWU | Male | 50 | Moderate | Anti-virus, TCM | 62 | 2 | 5mg, QD | 7 |  | 7 |  |
| 31 | ZHWU | Female | 55 | Moderate | Anti-virus, TCM | 33 | 2 | 5mg, QD | 7 |  | 9 |  |

HPPH: Henan Provincial Peoples Hospital

FAH: The First Affiliated Hospital of Henan University of Chinese Medicine

ZHWU: Zhongnan Hospital of Wuhan University

INFα: Interferon-α

Antiviral agents include: INFα, Chloroquine, Abidol, Ribavirin, Lopinavir/ritonavir, Ganciclovir, Favipiravir, Oseltamivir

**Table S4. MRM parameters for drug chemical analysis**

Table S4a. MRM parameters for FNC analysis.

| **Analytes** | **Polarity** | **Precursor ion (*m/z*)** | **Product ion (*m/z*)** | **Fragmentor（V）** | **CE (eV)** |
| --- | --- | --- | --- | --- | --- |
| FNC | + | 287 | 112 | 95 | 15 |
| FNC -NH+O | + | 288 | 113 | 95 | 15 |
| FNC -NH+O+CH2 | + | 302 | 127 | 95 | 15 |
| 3TC (IS) | + | 230 | 112 | 85 | 10 |
| FNC-MP | - | 364.9 | 158.8 | 145 | 30 |
| FNC-DP | - | 444.9 | 158.8 | 145 | 30 |
| FNC-TP | - | 524.9 | 158.8 | 145 | 30 |
| Succinate-d4 (IS) | - | 121.1 | 77.1 | 80 | 10 |

Table S4b. MRM parameters for remdexivir analysis

| **Analytes** | **Polarity** | **Precursor ion (*m/z*)** | **Product ion (*m/z*)** | **Fragmentor（V）** | **CE (eV)** |
| --- | --- | --- | --- | --- | --- |
| Remdexivir(GS-5734) | - | 601.1 | 328.1 | 145 | 15 |
| Nuc (GS-441524) | - | 290 | 175 | 105 | 15 |
| Nuc-MP | - | 370 | 97 | 125 | 20 |
| Nuc-TP(GS-441524) | - | 530 | 159 | 135 | 35 |
| Nuc-DP | - | 450 | 97 | 125 | 20 |
| Ala-Nuc (GS-704277) | - | 441 | 97 | 125 | 20 |
| 3TC (IS) | - | 227.9 | 133.9 | 95 | 15 |

**Table S5.** Reagents used in immunofluorescence analysis

|  | Name | Company | Product code |
| --- | --- | --- | --- |
| Rabbit Polyclonal Antibod  y | CD3 | Gene Tex | GTX42110 |
|  | CD20 | Abcam | Ab78237 |
|  | CD4 | Abcam | Ab133616 |
|  | CD8 | Abcam | Ab4055 |
|  | ACE2  S-Protein  N-Protein  IL-4  IL-10  RORγt  IL-13  Dapi | Abcam  Gene Tex  Sino Bio  Abcam  Gene Tex  Invitrogen  Invitrogen  Servicebio | Ab15348  GTX135356  MA14AP1502  Ab239508  GTX632359  14-6988-82  PA5-96053  G1012 |
| Goat anti rabbit IgG | HRP-conjugated | Servicebio | GB23301 |
| Fluorescent dye | Fitc-TSA  CY3-TSA  647-TSA | Servicebio Servicebio  Servicebio | G1222  G1223  G1224 |
